# Programming Injectable DNA Hydrogels Yields Tumor Microenvironment‐Activatable and Immune‐Instructive Depots for Augmented Chemo‐Immunotherapy

**DOI:** 10.1002/advs.202302119

**Published:** 2023-08-04

**Authors:** Yu Fan, Mengsi Zhan, Junhao Liang, Xingsen Yang, Beibei Zhang, Xiangyang Shi, Yong Hu

**Affiliations:** ^1^ Department of Polymeric Materials School of Materials Science and Engineering Tongji University Shanghai 201804 P. R. China; ^2^ College of Biological Science and Medical Engineering Donghua University Shanghai 201620 P. R. China

**Keywords:** chemo‐immunotherapy, DNA hydrogels, immune checkpoint blockade, immunogenic cell death, vaccine adjuvants

## Abstract

Injectable hydrogels have attracted increasing attention for promoting systemic antitumor immune response through the co‐delivery of chemotherapeutics and immunomodulators. However, the biosafety and bioactivity of conventional hydrogel depots are often impaired by insufficient possibilities for post‐gelling injection and means for biofunction integration. Here, an unprecedented injectable stimuli‐responsive immunomodulatory depot through programming a super‐soft DNA hydrogel adjuvant is reported. This hydrogel system encoded with adenosine triphosphate aptamers can be intratumorally injected in a gel formulation and then undergoes significant molecular conformation change to stimulate the distinct release kinetics of co‐encapsulated therapeutics. In this scenario, doxorubicin is first released to induce immunogenic cell death that intimately works together with the polymerized cytosine‐phosphate‐guanine oligodeoxynucleotide (CpG ODN) in gel scaffold for effectively recruiting and activating dendritic cells. The polymerized CpG ODN not only enhances tumor immunogenicity but minimizes free CpG‐induced splenomegaly. Furthermore, the subsequently released anti‐programmed cell death protein ligand 1 (aPDL1) blocks the corresponding immune inhibitory checkpoint molecule on tumor cells to sensitize antitumor T‐cell immunity. This work thus contributes to the first proof‐of‐concept demonstration of a programmable super‐soft DNA hydrogel system that perfectly matches the synergistic therapeutic modalities based on chemotherapeutic toxicity, in situ vaccination, and immune checkpoint blockade.

## Graphical abstract

An injectable DNA hydrogel with rationally programmed sequences and biofunctionalities is well established. This unprecedented system is made of repetitive adenosine triphosphate‐responsive aptamers and immunostimulatory cytosine‐phosphate‐guanine oligodeoxynucleotides, which can perfectly match the synergistic therapeutic modalities based on chemotherapeutic toxicity, in situ vaccination, and immune checkpoint blockade for effective suppression of tumor growth and lung metastasis.

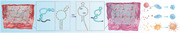

## 1 Introduction

With the development of cancer biology and immunology, immunotherapy has attracted tremendous attention and become a new approach for cancer treatment.^[^ ^1^, ^2^ ^]^ Based on the antitumor activities of cytotoxic T lymphocytes, immunotherapy shows high specificity and immune memory to tumor cells.^[^ ^3^, ^4^, ^5^ ^]^ In particular, blocking the interaction between programmed cell death 1(PD‐1) and programmed death‐ligand 1 (PD‐L1) with immune checkpoint inhibitors can significantly improve the durable responses and curative outcomes of cytotoxic T lymphocytes.^[^ ^6^ ^]^ However, the efficacy of immunotherapy with immune checkpoint inhibitors highly relies on the adaptive immune response. For another, chemotherapy with chemotherapeutics (e.g., doxorubicin [DOX], gemcitabine, oxaliplatin, and cyclophosphamide) is capable of triggering immune activation by inducing immunogenic cell death (ICD), activating antigen‐presenting cells, and improving T‐cell infiltration.^[^ ^7^, ^8^ ^]^ Unfortunately, chemotherapeutics were found to induce tumor immunosuppression by activating the overexpression of immunosuppression‐related genes such as PD‐L1.^[^ ^9^, ^10^ ^]^ Therefore, combination therapy of immune checkpoint blockade and immune activation promotion represents an advanced form of immunotherapy.

Injectable hydrogels have been investigated extensively to incorporate with drugs for tumor treatment because those materials can simultaneously deliver multiple drugs to the target sites with rational ratios and minimal invasion, and elevate their tumor accumulation, blood stability, and half‐lives.^[^ ^11^, ^12^, ^13^, ^14^ ^]^ Previous reports have shown that the mixture of hydrogel precursors could be injected into the tumor site, in which hydrogel scaffold formed for sequential release of chemotherapeutics and protein therapeutics, so as to elicit an immunogenic tumor phenotype and to promote immune‐mediated tumor regression.^[^ ^15^, ^16^ ^]^ Importantly, the local treatment of hydrogel‐encapsulated drugs showed superior tumor growth inhibition compared to the local or systemic delivery of non‐encapsulated drugs. Through further loading Toll‐like receptor (TLR) agonists (e.g., imiquimod, cytosine‐phosphate‐guanine oligodeoxynucleotide [CpG ODN]), injectable hydrogels could significantly amplify dendritic cell (DC) activation, tumor microenvironment (TME) inflammation, and tumor‐specific cytotoxic T lymphocyte responses.^[^ ^17^, ^18^ ^]^ However, since current studies regarding injectable chemo‐immunotherapeutic hydrogels are mainly based on the intratumoral gelation of soluble precursors, sol–gel transformation may be impeded by the complex physiological environment, resulting in the reduced cross‐linking degree, abrupt drug leakage, and unpredictable pharmacokinetics. Besides, conventional polymers lack molecular, structural, and functional programmability, which results in significant challenges in tailoring the biofunctionalities (e.g., immunomodulation, TME responsiveness) of hydrogel scaffolds for intimately interacting with tumor tissue.

DNA is a natural biopolymer with many advantages such as excellent biocompatibility, programmable diverse sequences, predictable secondary structure, precise self‐assembly process, and abundant biological stimulus‐response.^[^ ^19^, ^20^, ^21^ ^]^ Under favorable environments, DNA polymers can spontaneously self‐assemble into well‐defined hydrogels through either the ligation/hybridization of linear and branched DNA building blocks,^[^ ^22^, ^23^, ^24^ ^]^ or the enzymatic extension of oligonucleotides, in particular through rolling circle amplification (RCA).^[^ ^25^, ^26^, ^27^ ^]^ DNA hydrogels produced by the latter method represent a class of super‐soft metamaterials (elastic modulus <15 Pa), which display liquid‐like properties when taken out of water and solid‐like properties when immersed in water. In addition, the unique chemical features and porous structure of DNA hydrogels provide many interaction sites for proteins^[^ ^28^, ^29^ ^]^ and chemotherapeutics,^[^ ^30^, ^31^, ^32^ ^]^ enabling high loading efficiency and enhanced payload stability. Based on the molecular, structural, and functional information encoded on DNA building blocks, DNA hydrogels can be triggered by a variety of biomolecules, including adenosine triphosphate (ATP),^[^ ^33^ ^]^ glutathione,^[^ ^34^ ^]^ and enzyme,^[^ ^35^ ^]^ which is of particular importance for controlled drug release in target pathological tissues. In addition, through encoding CpG ODN on DNA scaffolds, the formed DNA hydrogel adjuvants can mimic the function of a lymph node, where the antigen‐presenting cells are recruited and activated by the high local concentration of CpG ODN.^[^ ^36^, ^37^ ^]^ Despite these advances, to the best of our knowledge, neither the design of TME‐responsive DNA hydrogel adjuvants with the feasibility of post‐gelling injection nor the investigation of their applications for co‐delivery of chemotherapeutics and immunomodulators is yet well developed.

**Figure 1:**
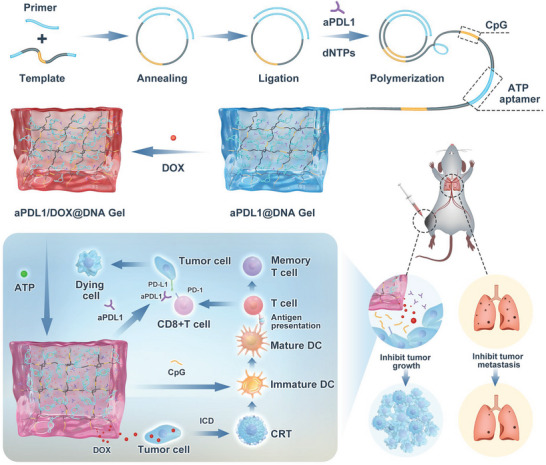
Schematics of the preparation of aPDL1/DOX@DNA Gel via RCA‐based polymerization for augmented chemo‐immunotherapy.

In this context, we describe DNA hydrogel adjuvants in which the repeats of CpG ODN and ATP aptamer are encoded on the ultralong DNA building blocks by RCA‐mediated DNA polymerization. By sequentially incorporating DNA hydrogel with immune checkpoint inhibitor aPDL1 and chemotherapeutic agent DOX, a chemo‐immunotherapeutic DNA hydrogel system (aPDL1/DOX@DNA Gel) is formed. Due to its super‐soft metaproperty, the DNA hydrogel system can be readily injected into tumor tissues in which the overexpressed ATP binds to the corresponding aptamer. This results in the conformational change of aptamer and volume expansion of the gel matrix, hence releasing the intercalated DOX to induce the ICD of tumor cells. Then the CpG ODN on the gel scaffold promotes the recruitment and maturation of DCs and the tumor infiltration of T cells by improving the immunogenicity of tumor‐associated antigens. The subsequently released aPDL1 antibody from the expanded gel matrix blocks the immune‐inhibitory PD‐L1 molecule on the tumor cell surface, thus sensitizing the tumor to antitumor T‐cell immunity (**Figure** **1**). This work provides an advanced strategy by simultaneously enhancing the tumor immunogenicity and reversing the immunosuppression to efficaciously attenuate the tumor growth, recurrence, and metastasis.

## 2 Results and Discussion

### 2.1 Synthesis and Characterization of DNA Gel

**Figure 2:**
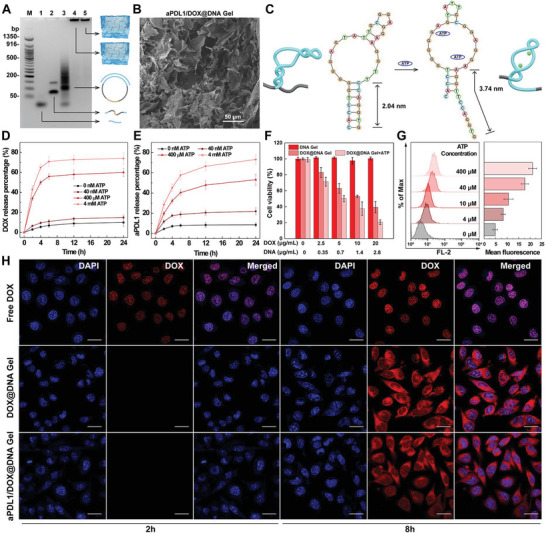
Characterization and drug release kinetics of as‐synthesized DNA Gel. A) Gel electrophoresis analyzing the circular template DNA and RCA products. Lane M: 50 bp DNA marker, lane 1: primer DNA, lane 2: linear template DNA, lane 3: circular template DNA, lane 4: RCA product, lane 5: RCA product loaded with aPDL1 antibody. B) SEM image of aPDL1/DOX@DNA Gel. C) Simulation of the conformational change of aptamer in the presence of ATP. Release kinetics of D) DOX and E) aPDL1 from DNA Gel with different ATP concentrations, respectively. F) Viabilities of B16 cells treated with DNA Gel, DOX@DNA Gel, and DOX@DNA Gel + ATP (400 µm) at different DNA or DOX concentrations for 24 h, respectively (*n* = 5). G) Flow cytometry analysis of fluorescence intensity in B16 cells treated with DOX@DNA Gel with different ATP concentrations (*n* = 3). H) Confocal microscopic images of DOX fluorescence in B16 cells pretreated with free DOX, DOX@DNA Gel, and aPDL1/DOX@DNA Gel for 2 and 8 h, respectively. Scale bar: 20 µm.

RCA is an isothermal amplification method that repeatedly copies a circular DNA template to produce an ultralong single‐stranded DNA with a periodic sequence.^[^ ^38^ ^]^ In this study, the DNA template was elaborately designed with complementary sequences of ATP aptamer and CpG ODN (Table S1, Supporting Information). To implement RCA polymerization, Phi29 DNA polymerase was used to elongate DNA primers on the enzymatically cyclized DNA templates and weave them non‐covalently into a hydrogel network. During polymerization, pyrophosphatase was used to simultaneously hydrolyze pyrophosphate, the byproduct of polymerization, inhibiting the formation of inorganic magnesium pyrophosphate crystals that are detrimental to the conformation of ATP aptamer (Figure S1, Supporting Information).^[^ ^39^ ^]^ The formation of circular DNA and ultralong DNA was verified by gel electrophoresis (**Figure** **2**A). Clearly, the circular DNA displayed slower migration than linear DNA, while the ultralong DNA was detained at the pockets without migration. Scanning electron microscopy (SEM) images reveal a typical 3D crosslinked network, suggesting the formation of DNA Gel (Figure 2B and Figure S2, Supporting Information). Next, dynamic rheology further verified the gel formation, because the storage modulus (*G*′) was constantly higher than the loss modulus (*G*″) in the tested frequency range (Figure S3, Supporting Information). Interestingly, DNA Gel not only exhibited an elastic modulus value as low as 10.3 Pa but could be stretched like a rubber band (Figure S4, Supporting Information). Those super‐soft metamaterial properties suggested the injectability of DNA Gel.^[^ ^32^ ^]^

### 2.2 Co‐Loading and Distinct Release of DOX and aPDL1

When aPDL1 antibody was used in the RCA reaction mixture, aPDL1 was physically encapsulated into a gel matrix through electrostatic interactions and magnesium‐mediated coordinative interactions between nucleic acids and proteins during polymerization,^[^ ^28^ ^]^ resulting in the formation of aPDL1@DNA Gel. By virtue of GC/CG‐rich stem as a drug association site in ATP aptamer,^[^ ^40^ ^]^ DNA Gel could be used for the enrichment of intercalating anthracycline DOX. Based on this, we prepared DOX‐loaded DNA Gel (DOX@DNA Gel) with different feeding ratios (Table S2 and Figure S5, Supporting Information). Given the relatively high drug loading efficiency (DLE) and loading content (DLC) of DOX, 20 µL of DOX solution and 50 µL DNA Gel or aPDL1@DNA Gel were selected to prepare DOX@DNA Gel and aPDL1/DOX@DNA Gel for subsequent studies, respectively. After that, the contents of DNA and aPDL1 were quantified using the commercial assay kits (Table S3, Supporting Information). The contents of aPDL1 in gels were estimated to be 9.3 g g^−1^ DNA for aPDL1@DNA Gel and 9.6 g g^−1^ DNA for aPDL1/DOX@DNA Gel, respectively. Meanwhile, the content of DOX in gels was estimated to be 7.2 g g^−1^ DNA for DOX@DNA Gel and 7.4 g g^−1^ DNA for aPDL1/DOX@DNA Gel, respectively. Then, the biostability of aPDL1/DOX@DNA Gel was investigated in both normal saline (NS) and DNase I solution. Clearly, the amount of aPDL1/DOX@DNA Gel after incubation in NS at 4 °C for 5 days remained nearly constant in all pockets of agarose gel, suggesting its excellent storability (Figure S6, Supporting Information). Interestingly, unlike DNA oligonucleotide that can be completely degraded within a few minutes, aPDL1/DOX@DNA Gel still maintained ≈50% integrity even after 12 h incubation with 5 U mL^−1^ DNase I (Figure S7, Supporting Information), which is considerably higher than the DNase I concentration in human blood (<1 U mL^−1^). The improved stability of DNA Gel can be attributed to the ultralong DNA building blocks and the highly crosslinked network, thereby impeding the nuclease accessibility.

Upon target binding, ATP aptamer indeed undergoes a significant conformational change, resulting in a decreased number of base pairs and an increased stem length (Figure 2C).^[^ ^41^ ^]^ To validate the ATP‐induced conformational change of aptamer in DNA Gel, the fluorescence spectra of DOX@DNA Gel before and after incubation with ATP were recorded. As shown in Figure S8, Supporting Information, the fluorescence intensity of DOX was significantly reduced by DNA Gel, which is attributed to the occurrence of donor‐quencher Förster resonance energy transfer (FRET) between DOX and aptamer.^[^ ^42^ ^]^ In contrast, the addition of ATP triggered the conformational change of aptamer, resulting in the prime release of DOX, subsequent FRET elimination, and ultimate fluorescence recovery. Besides, DNA Gel after incubation with ATP displayed a larger average pore size, as shown in SEM images (Figure S9, Supporting Information), and its whole volume was expanded to be 146% larger through glass tube volume expansion experiments. Therefore, in the presence of ATP, the intercalated drugs are supposed to dissociate from the aptamer stem and diffuse out of the gel matrix. Similarly, the intratumoral ATP‐induced conformational change of aptamer was validated by fluorescence imaging of tumors at 6 h post‐injection, where the ATP aptamer‐encoded DOX@DNA Gel displayed the stronger DOX fluorescence signal than DOX@DNA Gel without aptamer (Figure S10, Supporting Information), in agreement with the above‐mentioned results.

Then, the kinetics of stimuli‐responsive drug release from aPDL1/DOX@DNA Gel were investigated in TME mimetic solutions (Figure 2D and Figure S11, Supporting Information).^[^ ^43^ ^]^ It was found that the release amount of DOX from gel matrix in different solutions follows the order of TME mimetic solution (intracellular: 4 mm ATP, 74 %; extracellular: 400 µm ATP, 60%) > physiologically mimetic solution (40 nm ATP, 15 %) > ATP‐free solution (11%) at 24 h. In contrast, less than 10% DOX was released under both physiologic pH (7.4) and TME pH (6.8) within 24 h (Figure S12, Supporting Information). These results suggest that ATP aptamer‐encoded DNA Gel displayed pH‐independent but rather ATP‐dependent drug release kinetics. Similarly, the release amount of aPDL1 antibody was positively correlated with the concentration of ATP (4 mm ATP, 73%; 400 µm ATP, 53%; 40 nm, 22%; 0 nm, 9% at 24 h) (Figure 2E). These results suggest that aPDL1/DOX@DNA Gel with ATP‐responsiveness provides a promising platform for efficient chemo‐immunotherapy of tumors. Interestingly, most of DOX was released from the gel matrix after incubation in TME mimetic solutions for 6 h, whereas aPDL1 displayed a sustained release profile within 24 h. It is believed that the distinct release kinetics are important for intended sequential chemotherapeutic and immunotherapeutic effects.^[^ ^15^ ^]^ Furthermore, the drug release profiles in the presence of nuclease were investigated. Clearly, DNase I stimulated the inconspicuous release of DOX and aPDL1 during the entire study period due to the exceptional nuclease resistance of DNA Gel (Figure S13, Supporting Information).

Then, cell viability assays were carried out to compare the cytotoxicity of free DOX and intercalated DOX in DNA Gel to B16 cancer cells (a murine melanoma cell line). As shown in Figure 2F, DNA Gel clearly showed negligible inhibition effect on cell proliferation and thus proved its good cytocompatibility, whereas DOX@DNA Gel showed a concentration‐dependent inhibition on B16 cells similar to free DOX (Figure S14, Supporting Information), and the viability of B16 cells decreased to 53.1% after 24 h treatment of DOX@DNA Gel with DOX concentration of 10 µg mL^−1^. Interestingly, the killing efficiency of DOX@DNA Gel could be enhanced by ATP (400 µm), which is due to the extracellular ATP‐triggered release of DOX from the gel matrix. In order to monitor the intracellular DOX, flow cytometry was used to examine B16 cells treated with DOX@DNA Gel. The results showed that the treated cells exhibited ATP concentration‐dependent DOX fluorescence intensity (Figure 2G). The DOX fluorescence intensity of cells treated with DOX@DNA Gel+ATP at the ATP concentration of 10, 40, or 400 µm is about 2.2, 3.6, or 4.3 times higher than that of cells treated with DOX@DNA Gel only. Apparently, the presence of ATP elevated the intracellular DOX accumulation and subsequently induced higher cytotoxicity (Figure 2H). The intracellular DOX accumulation was further visualized through confocal laser scanning microscopy. As shown in Figure 2G, the nuclei of B16 cells treated with free DOX displayed obvious fluorescence signals within 2 h, while B16 cells treated with free DOX or DOX@DNA Gel for 8 h displayed comparable fluorescence intensities. Besides, the loading of aPDL1 did not affect the intracellular DOX accumulation. Collectively, the successive extracellular and intracellular DOX release pathways and the proven cytotoxicity of released DOX under TME mimetic environments ensure the availability of DOX‐loaded DNA Gels for efficient chemotherapy.

### 2.3 In Vitro Maturation of DCs

**Figure 3:**
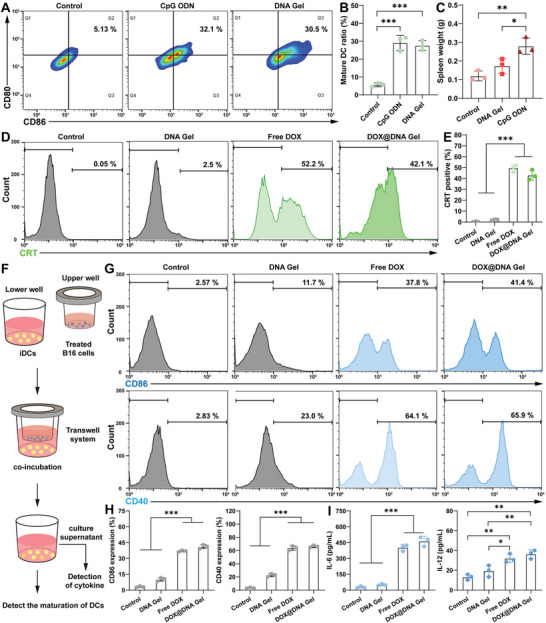
In vitro maturation of DCs and in vivo spleen compatibility. A) Flow cytometry analysis of the expression of costimulatory marker CD80/86 on DCs after treatment with free CpG ODN and DNA Gel, respectively. B) The statistical analysis of DC maturation according to flow cytometry analysis. C) The statistical analysis of the spleen weights of mice after treatment with CpG ODN and DNA Gel, respectively. D) Flow cytometry analysis of CRT exposure on B16 cells after treatment with DNA Gel, free DOX, and DOX@DNA Gel, respectively. E) The statistical analysis of CRT‐positive B16 cells according to flow cytometry analysis. F) Schematic illustration of transwell system experiment for maturation of DCs. G) Flow cytometry analysis and H) statistical analysis of the expression of CD86/40 on DCs after 24 h co‐culture with B16 cells pretreated with DNA Gel, free DOX, and DOX@DNA Gel, respectively. I) Secretion levels of IL‐6 and IL‐12 in the supernatant of DCs after 24 h co‐culture with B16 cells pretreated with DNA Gel, free DOX, and DOX@DNA Gel, respectively. (*n* = 3).

Unmethylated CpG ODN, a TLR9 agonist, can trigger innate and adaptive immunity and induce cytokine secretion by maturation of DCs.^[^ ^44^ ^]^ To investigate the maturation of DCs by the polymerized CpG ODN in DNA Gel, DCs were incubated with DNA Gel for 24 h before flow cytometry analysis of costimulatory markers (e.g., CD86 and CD80) related to DC maturation (**Figure** **3**A,B). Clearly, the expressions of CD86 and CD80 on DCs were greatly improved after incubation with either DNA Gel or free CpG ODN, suggesting the intense DC maturation by DNA Gel. Furthermore, cytokine concentrations in the supernatant of DCs were measured (Figure S15, Supporting Information). The concentrations of IL‐6 and IL‐12 in the culture supernatant of DNA Gel treated DCs (IL‐6: 230.9 ± 26.6 pg mL^−1^; IL‐12: 34.3 ± 3.2 pg mL^−1^) were higher than those in the culture supernatant of CpG ODN treated DCs (IL‐6: 176.7 ± 15.2 pg mL^−1^, IL‐12: 22.6 ± 2.3 pg mL^−1^). The higher levels of cytokines in the DNA Gel group were most likely due to that the polymerized CpG ODN displayed improved nuclease resistance than free CpG ODN.^[^ ^45^ ^]^ All these findings indicated that DNA Gel could serve as an immunoadjuvant to induce the expression of costimulatory molecules for the maturation of DCs, and to improve the secretion of proinflammatory cytokines to further elevate the maturity of DCs.

Due to the short tissue retention and fast systemic circulation, one of the main side effects of conventional CpG ODN is splenomegaly associated with extramedullary hematopoiesis.^[^ ^46^, ^47^ ^]^ To evaluate if the polymerized CpG ODN can attenuate this side effect, DNA Gel or free CpG ODN was subcutaneously injected into mice, followed by determination of spleen weight to assess splenomegaly (Figure S16, Supporting Information). After treatment with free CpG ODN for 3 days, mice suffered from transient splenomegaly with a 2.3‐fold increase in spleen weight. Importantly, DNA Gel treated mice did not show obvious splenomegaly compared to untreated mice, which clearly indicates the reduction of systemic side effects by polymerization of CpG ODN in DNA Gel (Figure 3C and Figure S17, Supporting Information). We inferred that DNA Gel displayed excellent spleen compatibility by rendering CpG ODN prolonged tissue retention time and reduced systemic circulation.^[^ ^48^ ^]^ To validate this hypothesis, we further examined the biodistribution of Cy3‐labeled CpG ODN and Cy3‐labeled DNA Gel via fluorescence imaging. As depicted in Figure S18, Supporting Information, free CpG ODN displayed very low tumor retention and its majority drained into systemic circulation at 6 h post‐treatment. In sharp contrast, DNA Gel displayed very high tumor retention with a significant reduction of the systemic circulation, which is attributed to the restricted in vivo motility and metabolism of the polymerized CpG ODN, thereby reducing the adverse effects of CpG ODN while enhancing its tumor immunomodulatory efficacy.

Furthermore, the biocompatibility of DNA Gel was assessed by hematoxylin and eosin (H&E) staining of major organs and serum biochemistry marker analysis. Apparently, there were no obvious cytotoxicity or inflammatory infiltrates in the major organs of mice treated with DNA Gel (Figure S19, Supporting Information). Else, the liver function parameters (Figure S20, Supporting Information, alanine aminotransferase, ALT; aspartate aminotransferase, AST), as well as kidney function parameters (Figure S21, Supporting Information, creatinine, CREA; Blood urea nitrogen, BUN) of mice treated with DNA Gel remained at normal levels similar to the those of untreated mice.

Next, we further investigated whether DOX@DNA Gel could induce ICD of B16 cells. Flow cytometry results showed a significantly higher level of calreticulin (CRT) expression on either free DOX‐treated cells (49.6%) or DOX@DNA Gel treated cells (43%) compared to DNA Gel treated cells (2.1%) and untreated cells (0.3%) (Figure 3D,E). Rapid translocation of CRT to the cell surface can serve as an “eat me” signal to antigen‐presenting cells, leading to immunogenic uptake of tumor antigens and subsequent generation of antigen‐specific T cell responses.^[^ ^49^ ^]^ To examine the ICD‐mediated DC maturation, a transwell culture system was adopted to co‐culture DCs with pre‐treated B16 cells (Figure 3F). After treatment with DNA Gel, free DOX, or DOX@DNA Gel, B16 cells were placed in the upper wells and incubated with DCs in the lower basal compartment for 24 h. We observed significant upregulation of the costimulatory molecules CD86 and CD40 on DCs after co‐culture with cancer cells pretreated with both free DOX and DOX@DNA Gel compared with DCs after co‐culture with the untreated cancer cells (Figure 3G,H). Furthermore, the secretion of IL‐6 and IL‐12 by matured DCs was investigated to delineate the potentiated downstream activation of T cells (Figure 3I).^[^ ^1^ ^]^ Compared to DCs after co‐culture with the untreated cancer cells and DNA Gel treated cancer cells, DCs after co‐culture with cancer cells pretreated with both free DOX and DOX@DNA Gel secreted higher doses of IL‐6 and IL‐12 (*p*<0.01). Our data suggest that DOX@DNA Gel can effectively elicit antitumor immune responses by inducing the ICD of tumor cells in addition to killing tumor cells directly.

### 2.4 Chemo‐Immunotherapy of Primary Tumors

**Figure 4:**
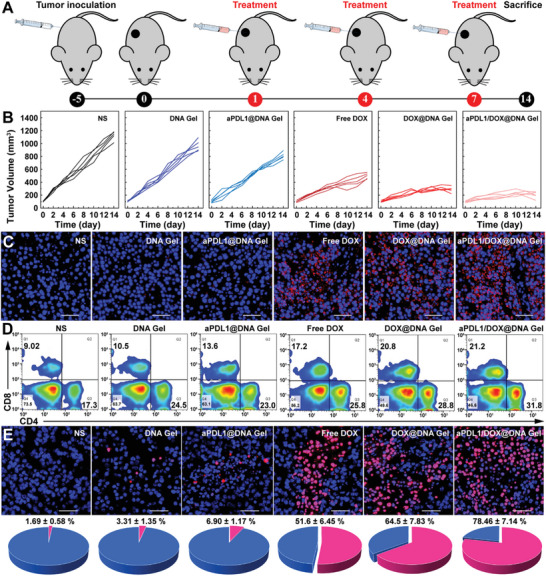
In vivo chemo‐immunotherapy of primary tumors. A) Schematic illustrating chemo‐immunotherapy of a murine melanoma model with aPDL1/DOX@DNA Gel. B) Individual tumor growth curves for the indicated groups (*n* = 5). C) Immunohistochemical staining of CRT in the tumor sections after indicated treatments. Scale bars: 50 µm. D) Flow cytometry results showing the proportions of CD4+ and CD8+ T cells in the tumors of different groups. E) TUNEL staining of the tumor sections after indicated treatments. Scale bars: 50 µm. Pie charts show distributions of normal (blue) and apoptotic (red) tumor cells, respectively.

In vivo therapeutic efficacy of aPDL1/DOX@DNA Gel was evaluated using a murine melanoma model according to the treatment protocol shown in **Figure** **4**A. It was found that free DOX, DNA Gel, and aPDL1@DNA Gel displayed a moderate tumor suppression effect as compared to NS (Figure 4B and Figure S22, Supporting Information). This suggests that single‐mode chemotherapy or immunotherapy is unable to exert desired antitumor effect due to the high degree of malignancy and limited immune modulation.^[^ ^50^ ^]^ In contrast, the synergy of chemotherapy and adjuvant immunotherapy using DOX@DNA Gel displayed significant antitumor efficacy, which even could be further improved with additional immune checkpoint blockade using aPDL1/DOX@DNA Gel. In addition, there were no significant changes in the body weight of treated mice, suggesting the biocompatibility of hydrogels (Figure S23, Supporting Information). Then, immunohistochemical staining was conducted for examining the expression level of ICD markers on tumor cells. As shown in Figure 4C and Figure S24, Supporting Information, aPDL1/DOX@DNA Gel treatment induced the highest level of CRT exposure in tumor cells among different treatments. To explore the amplified tumor immunogenicity by CpG ODN, we determined the concentrations of immune‐related cytokines in blood serum (Figure S25, Supporting Information). Clearly, the contents of IL‐6 and IL‐12 in the aPDL1/DOX@DNA Gel group are much higher than other groups (*p* < 0.01), and follow the order of aPDL1/DOX@DNA Gel > DOX@DNA Gel > free DOX > aPDL1@DNA Gel > DNA Gel > NS. These results are consistent with the findings of in vitro DC maturation (Figure 3I). To further confirm the activation of T‐cell immunity, the tumor‐infiltrating T cells were extracted and analyzed by flow cytometry. Obviously, the treatment of aPDL1/DOX@DNA Gel significantly increased the populations of infiltrated CD4+ and CD8+ T cells compared with other treatments (Figure 4D and Figure S26, Supporting Information). Therefore, these results suggest that the immune responses induced by aPDL1/DOX@DNA Gel can up‐regulate the percentages of tumor‐infiltrating effector T cells and activate a large proportion of cytotoxic T cells in tumors for restraining tumor growth.^[^ ^2^ ^]^ Then, tumor tissues were further examined via H&E staining and TdT‐mediated dUTP Nick‐End Labeling (TUNEL) staining. As illustrated in Figure 4E and Figure S27, Supporting Information, the highest levels of necrotic (H&E, ≈26%) and apoptotic (TUNEL, ≈78%) cells were found in the aPDL1/DOX@DNA Gel treated tumors, demonstrating the enhanced anti‐tumor effect of synergistic chemo‐immunotherapy. Collectively, these results clearly suggest that aPDL1/DOX@DNA Gel exhibits potent efficacy of chemo‐immunotherapy through DOX‐mediated chemotherapy and synergistic modulation of immune response by ICD effect, CpG immunoadjuvant, and immune checkpoint inhibitor aPDL1 antibody.

### 2.5 Inhibition of Lung Metastasis by Immune Memory

**Figure 5:**
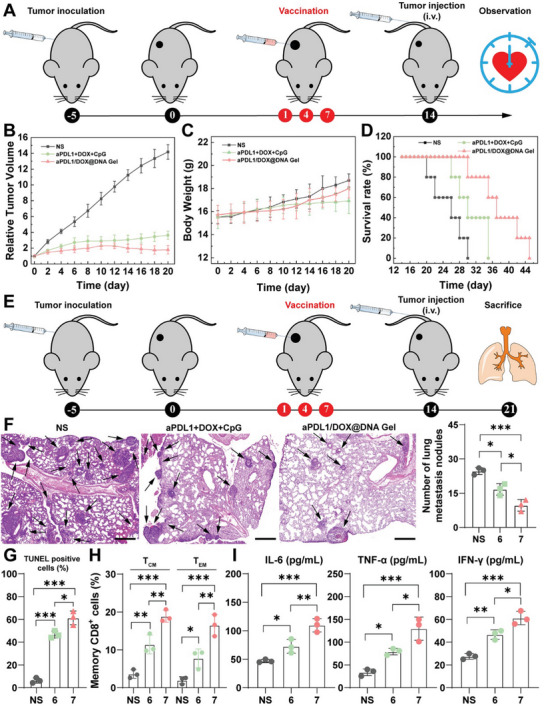
In vivo anti‐metastasis and immune memory effect. A) Schematic illustrating chemo‐immunotherapy of a metastatic melanoma model with aPDL1/DOX@DNA Gel. B) Relative tumor volumes, C) body weights, and D) survival rates of B16 tumor‐bearing mice with lung metastasis after different treatments. E) Schematic illustrating prevention of lung metastasis via immune memory effect. F) Histological images and statistical analysis of lung metastatic foci of mice after different treatments. Metastatic foci are indicated with a black arrow. Scale bars: 500 µm. G) The statistical analysis of TUNEL‐positive cells in tumor tissue sections of mice after various treatments. H) The statistical analysis of T_CM_ and T_EM_ cells in spleens of mice after different treatments (*n* = 3). I) The statistical analysis of IL‐6, TNF‐α, and IFN‐γ in the blood serum of mice after different treatments. Group 6: aPDL1+DOX+CpG; Group 7: aPDL1/DOX@DNA Gel.

Encouraged by the strong growth inhibition and immunostimulation of primary tumors by aPDL1/DOX@DNA Gel, the well‐established bloodstream metastasis model was further utilized to evaluate the tumor inhibition, anti‐metastasis, and immune memory effect. As shown in **Figure** **5**A, B16 tumor‐bearing mice were intratumorally injected with aPDL1/DOX@DNA Gel or aPDL1+DOX+CpG, respectively, for a total of three times. On the 14th day after treatment, tumor‐bearing mice were intravenously re‐challenged with B16 cells. As shown in Figure 5B and Figure S28, Supporting Information, the volume change of tumors reveals that both aPDL1/DOX@DNA Gel and aPDL1+DOX+CpG can significantly suppress tumor growth. Nevertheless, the anti‐tumor efficacy of aPDL1/DOX@DNA Gel is superior to aPDL1+DOX+CpG. Notably, during the treatment period, no obvious body weight loss was detected in all groups, indicating low systemic toxicity of hydrogels (Figure 5C). Besides, 100% of mice in the aPDL1/DOX@DNA Gel group survived for a time period of 30 days, while 100% of mice in the NS group and 60% of mice in the aPDL1+DOX+DNA Gel group were dead (Figure 5D). These results suggest that aPDL1/DOX@DNA Gel can effectively inhibit tumor growth and prolong the survival time of a metastatic melanoma model.

In parallel experiments, metastatic melanoma mice after different treatments were sacrificed for analysis of necrotic and apoptotic cells in tumors, metastatic foci in the lung, T_CM_ and T_EM_ cells in the spleen, and cytokines in blood serum at 21 days post‐treatment (Figure 5E). First, we analyzed the tumor tissue sections via H&E staining and TUNEL staining. As illustrated in Figure S29, Supporting Information, the highest levels of necrotic (H&E) and apoptotic (TUNEL) cells in the tumors were observed in aPDL1/DOX@DNA Gel group. The cell apoptosis rate was statistically analyzed in different groups, which follows the order of aPDL1/DOX@DNA Gel (61.4 ± 6.1%) > aPDL1+DOX+CpG (46.7 ± 3.1%) > NS (6.3 ± 2.3%), demonstrating the most effective anti‐cancer effect of aPDL1/DOX@DNA Gel (Figure 5G). We then investigated the lung metastatic foci in the metastatic melanoma mice after different treatments (Figure 5F and Figures S30 and S31, Supporting Information). An abundance of nodules (nuclei‐rich, deeper‐stained regions) were found in the lungs extracted from mice in the NS group, while a decreased number of nodules were observed in the aPDL1+DOX+CpG group. Importantly, aPDL1/DOX@DNA Gel was able to induce an anti‐metastatic effect to a much greater extent than aPDL1+DOX+CpG (*p*<0.05). To understand the fundamental immune mechanisms behind the anti‐metastasis, we then determined the proportions of memory T cells in the spleen and doses of proinflammatory cytokines in blood serum in different groups. To this end, T cells in spleens were extracted to evaluate the proportions of central memory T cells (T_CM_) and effector memory T cells (T_EM_) after different treatments. As shown in Figure 5H and Figures S32–S34, Supporting Information, the percentages of splenic T_CM_ and T_EM_ cells in the aPDL1/DOX@DNA Gel group were much higher than in other groups (*p* < 0.01). Meanwhile, the levels of proinflammatory cytokine IL‐6, tumor‐necrosis factor‐α (TNF‐α), and interferon‐γ (IFN‐γ) were detected in the blood serum of different groups, and the results showed that the levels of all those cytokines in aPDL1/DOX@DNA Gel group were significantly higher than other groups (Figure 5I).

To evaluate the biocompatibility of aPDL1/DOX@DNA Gel, the major organs (e.g., heart, liver, spleen, and kidney) of metastatic melanoma mice after different treatments were extracted for sectioning, followed by examination via H&E staining. Clearly, there were no evident physiological morphological changes in the major organs of aPDL1/DOX@DNA Gel‐treated mice, compared to NS‐treated mice (Figure S35, Supporting Information). Furthermore, biochemical analysis of the blood serum in the aPDL1/DOX@DNA Gel group confirmed no evident changes in liver function (ALT and AST) and kidney function (CREA and BUN) versus the NS group (Figure S36, Supporting Information). All these results suggest the excellent biocompatibility of chemo‐immunotherapeutic aPDL1/DOX@DNA Gel.

## 3 Conclusion

In summary, an injectable DNA hydrogel was programmed as a TME‐activatable and immune‐instructive depot for augmented chemo‐immunotherapy. After intratumoral injection, aPDL1/DOX@DNA Gel with the encoded ATP aptamers underwent significant molecular conformation change upon target binding, eventually resulting in the volume expansion that facilitated the distinct release kinetics of co‐encapsulated therapeutics. Thereafter, DOX was first released to directly kill tumor cells and meanwhile to induce the ICD of tumor cells, followed by working together with the polymerized CpG ODN for effectively recruiting and activating DCs. Importantly, the polymerized CpG ODN could enhance the tumor immunogenicity and meanwhile minimize splenomegaly, the side effect of free CpG ODN. Furthermore, aPDL1 antibody was subsequently released from hydrogel to block the immune inhibitory checkpoint molecules PD‐L1 on the tumor cell surface, thus reversing the TME immunosuppression through potentiating T‐cell mediated immune responses. The programmed aPDL1/DOX@DNA Gel demonstrated potent suppression of tumor growth and lung metastasis via the induced strong systemic immune response and immune memory effect, thanks to perfectly matching the sequence programmability to the synergistic therapeutic modalities based on chemotherapeutic toxicity, in situ vaccination, and immune checkpoint blockade. With the high programmability of design principle, we, therefore, believe that our approach can be combined with DNA‐protein conjugation chemistry,^[^ ^29^ ^]^ DNA nanotechnology,^[^ ^20^ ^]^ and microfluidic technique^[^ ^24^ ^]^ to develop a library of biomaterial systems in new biomedical applications beyond chemo‐immunotherapy of tumors.

## 4 Experimental Section

### Preparation of Circular Template DNA

To serve as a suitable template for RCA, linear template DNA was circularized by ligation with T4 DNA ligase. Briefly, 5 µL of 5′‐phosphorylated linear DNA (100 µm) was hybridized with 10 µL of primer (100 µm) in ligase reaction buffer (50 mm Tris‐HCl, 10 mm MgCl_2_, 1 mm ATP, 10 mm dithiothreitol [DTT], pH 7.5) by heating at 95 °C for 5 min and slowly cooling to room temperature for at least 3 h. Ligation was then performed by adding 5 µL of T4 DNA ligase (400 U µL^−1^), followed by incubation at 16 °C overnight. The enzyme was inactivated by heating at 65 °C for 10 min. The resulting circular template DNA was confirmed by gel electrophoresis analysis in TBE buffer (89 mm Tris‐HCl, 89 mm boric acid, 2 mm EDTA, pH 8.3). To visualize DNA, the gels were stained with ethidium bromide at room temperature for 30 min and photographed by the GenoSens 2100 Gel Imaging System after the electrophoresis.

### Preparation of DNA Gel, aPDL1@DNA Gel, and aPDL1/DOX@DNA Gel

To synthesize aPDL1‐loaded DNA Gel, RCA was employed using single‐stranded primer DNA and circular template DNA in the presence of aPDL1. Briefly, each reaction was conducted in a final volume of 50 µL consisting of circular template DNA (2 µm, 15 µL), dNTPs (10 mm, 5 µL), aPDL1 solution (25 mg mL^−1^, 2 µL), pyrophosphatase (PPase, 1 µL, 0.1 U mL^−1^), and Phi29 DNA polymerase (5 µL, 10 U µL^−1^) in RCA reaction buffer (50 mm Tris‐HCl, 10 mm MgCl_2_, 10 mm (NH_4_)_2_SO_4_, 4 mm DTT, pH 7.5). The reaction mixtures were incubated at 30 °C for 48 h. Nuclease‐free water (50 µL) was added to the sample for dilution and then the RCA products were washed with nuclease‐free water three times. The obtained hydrogels were kept in nuclease‐free water at 4 °C. As a control, DNA Gel was synthesized via the same procedures in the absence of aPDL1. To prepare aPDL1/DOX@DNA Gel, different volumes of DOX stock solution (2 mg mL^−1^) were mixed with DNA Gels (50 µL), respectively, and water was added to reach a final volume of 100 µL. The mixtures were incubated overnight. A full description of experimental procedures can be found in Supporting Information.

### In Vitro Release Kinetics

The release of DOX and aPDL1 from DNA Gel was studied under different conditions. In a typical experiment, 100 µL of DOX@DNA Gel or aPDL1@DNA Gel was added to 100 µL of PBS (pH 7.4 or 6.8), or ATP solution (40 nm, 400 µm or 4 mm), respectively, and incubated with 200 rpm orbital shaking for variable times. The supernatant was then collected for the quantitative analysis of released DOX by UV–vis spectroscopy at 480 nm and released aPDL1 using NanoOrange Protein Kit. The volume of the medium sample was maintained constant by replenishing the corresponding solution. The experiment was performed in triplicate for each sample.

### Cell Culture and In Vitro Assays

B16 or DC cells were regularly cultured, passaged, and adopted for in vitro experiments including CCK‐8 cytotoxicity assay, cellular uptake, flow cytometry, confocal microscopic observation, and assays of ICD and maturation of DCs.

### Animal Experiments

All animal experiments were carried out following the protocols approved by the Animal Care and Use Committee of Tongji University and also in accordance with the policy of the National Ministry of Health of China. Healthy C57BL/6 mice were used to evaluate the in vivo biocompatibility of the DNA Gel. B16 xenografted tumor model was established to evaluate the in vivo anti‐tumor therapeutic efficacy of aPDL1/DOX@DNA Gel. A B16 bloodstream metastasis model was established to evaluate the anti‐metastasis effect of aPDL1/DOX@DNA Gel. A full description of experimental procedures can be found in the Supporting Information.

## Conflict of Interest

The authors declare no conflict of interest.

## Supporting information

Supporting Information

## Data Availability

The data that support the findings of this study are available from the corresponding author upon reasonable request.
