# Supplementary material for: Programming Injectable DNA Hydrogels Yields Tumor Microenvironment‐Activatable and Immune‐Instructive Depots for Augmented Chemo‐Immunotherapy
Source: Adv Sci (Weinh). 2023 Aug 4;10(29):2302119. doi: 10.1002/advs.202302119 (PMC10582419; doi:10.1002/advs.202302119)
Supplement: Supplementary file 1 — Supporting Information [file ADVS-10-2302119-s001.pdf]

ADVANCED SCIENCE
Open Access

Supporting Information

for *Adv. Sci.*, DOI 10.1002/advs.202302119

Programming Injectable DNA Hydrogels Yields Tumor Microenvironment-Activatable and Immune-Instructive Depots for Augmented Chemo-Immunotherapy

Yu Fan, Mengsi Zhan, Junhao Liang, Xingsen Yang, Beibei Zhang, Xiangyang Shi and Yong Hu**

Supporting Information

Programming Injectable DNA Hydrogels Yields Tumor Microenvironment-Activatable and Immune-Instructive Depots for Augmented Chemo-Immunotherapy

Yu Fan, Mengsi Zhan, Junhao Liang, Xingsen Yang, Beibei Zhang, Xiangyang Shi, and Yong Hu**

Dr. Y. Fan, J. H. Liang, X. S. Yang, B. B. Zhang, Prof. Y. Hu
Department of Polymeric Materials, School of Materials Science and Engineering, Tongji University, Shanghai 201804, P. R. China.
E-mail: yonghu@tongji.edu.cn (Y. Hu)

M. S. Zhan, Prof. X. Y. Shi
College of Biological Science and Medical Engineering, Donghua University, Shanghai 201620, P. R. China
E-mail: xshi@dhu.edu.cn (X. Shi)

Keywords: DNA hydrogels; vaccine adjuvants; immunogenic cell death; immune checkpoint blockade; chemo-immunotherapy

Experimental Section

Materials. HPLC-purified oligonucleotides were purchased from Sangon Biotech Co., Ltd. (Shanghai, China). T4 DNA ligase, T4 buffer solution, phi29 DNA polymerase, Phi29 buffer solution, and deoxynucleotides (dNTPs) solution mix were obtained from New England Biolabs (UK). Agarose, ATP Solution (10 mM), DNA ladder, loading dye, PicoGreen dsDNA assay Kit and all ELISA kits were obtained from Thermo Fisher Scientific. Doxorubicin hydrochloride (DOX) was from Beijing HVSF United Chemical Materials Co., Ltd. (Beijing, China). Programmed cell death ligand 1 antibody (aPDL1) was purchased from Bio X Cell (West Lebanon, NH). RNase/DNase-free distilled water (Thermo Fisher Scientific) was used for the preparation of all aqueous solutions and RNase/DNase-free Eppendorf microcentrifuge tubes were used for DNA ligation and polymerization. Cell counting kit-8 (CCK-8) was from Beyotime Biotechnology Co., Ltd. (Shanghai, China). B16 cells (a murine melanoma cell line) were acquired from Institute of Biochemistry and Cell Biology (the Chinese Academy of Sciences, Shanghai, China). Dendritic cells (DCs) were acquired from Shanghai Cancer Center of Fudan University. Roswell Park Memorial Institute (RPMI)-1640 medium and Dulbecco's Modified Eagle Medium (DMEM) were from HyClone Lab., Inc. (Logan, UT). Fetal bovine serum (FBS) was from Gibco (Carlsbad, CA). Penicillin-streptomycin and trypsin (0.25%) were purchased from Gino Biomedical Technology Co., Ltd. (Hangzhou, China). Tumor infiltrating lymphocyte cell separation medium kit (mice) was from Beijing Solarbio Science & Technology Co., Ltd. (Beijing, China). The anti-CD80-FITC, anti-CD86-PE, anti-CD40-FITC, anti-CD3-FITC, anti-CD4-APC, anti-CD8-PE, anti-CD44-FITC, and anti-CD62L-APC for flow cytometry assays were obtained from Biolegend (San Diego, CA).

Preparation of aPDL1/DOX@DNA Gel. Different volumes of DOX stock solution (2 mg/mL) were mixed with DNA Gels (50 μL) respectively, and water was added to reach final volume of 100 μL.

The mixtures were incubated overnight. The unbound DOX in the supernatant was collected carefully and measured *via* UV-vis spectroscopy to quantify the amount of intercalated DOX. Then, DOX-loaded DNA Gels were washed several times to remove the unbound DOX. The drug-loading content and drug encapsulation efficiency were calculated as below:

$$\text{Drug loading content (\%)} = W_t / W_s \times 100\% \quad (1)$$

$$\text{Encapsulation efficiency (\%)} = W_t / W_0 \times 100\% \quad (2)$$

where W_t represents the mass of loaded DOX within the DNA Gel, W_0 represents the initial amount of DOX; W_s represents the mass of DOX-loaded DNA Gel.

Characterization Techniques. For SEM imaging, all samples were lyophilized using the laboratory lyophilizer, and then placed onto the sample stage using conductive adhesive. Finally, the samples were metal-coated with Au for SEM (Hitachi-S4800 FESEM).

The amounts of DNA and protein in DNA Gel were quantified using PicoGreen dsDNA reagent and NanoOrange Protein Kit, respectively. Briefly, different DNA Gels were incubated with the PicoGreen reagent in a 96-well solid black microplate for 5 min at room temperature and the fluorescence intensity (Ex = 480 nm, Em = 525 nm) was measured using an EnVision multilabel plate reader. The DNA concentration was determined according to the standard curve of serial dilutions of lambda DNA (λ-DNA) provided by the manufacturer. The same samples were further incubated with NanoOrange working solution at 95°C for 10 minutes (protected from light), then cooled to room temperature in a dark drawer. The protein concentration was determined by the fluorescence intensity (Ex = 485 nm, Em = 590 nm). The standard curve was prepared using serial concentrations of aPDL1 solutions.

The rheological properties were carried out on a HR-2 rheometer (TA Instruments) equipped. The test was performed in an 8 mm parallel-plate geometry using 100 μL DNA Gel. The frequency sweep test was performed at 25 °C with a fixed strain (1%).

ATP Responsiveness Assays. To investigate ATP responsiveness, four main experiments were conducted both in vitro and in vivo, which were listed as the following:

Firstly, 100 μL DNA Gel was transferred into a glass tube with inner diameter of 4 mm. The length of gel in tube was measured before (L_0) and after (L) incubation with ATP (400 μM, 50 μL) for 5 h. The volume expansion ratio of hydrogel was calculated using the following formula:

Volume expansion ratio = $L / L_0 \times 100\%$ (3)

Secondly, the pore size change of DNA Gel after incubation with ATP was visualized by SEM. Briefly, 50 μL DNA Gel was incubated with ATP (400 μM) for 5 h. Prior to SEM imaging, DNA Gel was subjected to lyophilization. DNA Gel without ATP treatment was used as control.

Thirdly, the donor-quencher FRET between DOX and aptamer was used to study the aptamer conformational change induced by ATP. In brief, the fluorescence spectra of DOX@DNA Gel (50 μL) before and after incubation with ATP (400 μM) for 5 h were recorded at Ex = 480 nm.

Fourthly, the intratumoral ATP-induced conformational change of aptamer was further investigated. Specifically, B16 tumor-bearing mice were intratumorally administered with 100 μL of ATP-responsive DOX@DNA Gel and non-responsive DOX@DNA Gel, respectively. After 6 h, the tumors were extracted from mice and then imaged on a commercial VISQUE *In Vivo* Smart Imaging System. Of note, ATP-responsive DOX@DNA Gel and non-responsive DOX@DNA Gel were synthesized via RCA of Primer on Template and Primer-1 on Template-1, respectively. Unless otherwise stated, ATP-responsive DNA Gel was used.

Cell Culture and Cytotoxicity Assays. B16 cells were regularly cultured in RPMI-1640 medium containing 10% FBS and 1% penicillin-streptomycin in a Thermo Scientific cell incubator (Waltham, MA) at 5% CO_2 and 37 °C. For cell counting kit-8 (CCK-8) assay, cells were seeded in 96 well plates at a density of 1×10^4 cells per well with 100 μL medium for each well and incubated overnight. After that, the medium in each well was replaced with fresh medium containing DOX at various concentrations and the cells were incubated for 24 h. Then the cells were washed with PBS and treated with 100 μL serum-free medium containing 10% CCK-8 for additional 2 h. Finally, the absorbance of each well was recorded by a Thermo Scientific Multiskan MK3 ELISA reader (Waltham, MA) at 450 nm. Each sample was tested performed in sextuplicate. The cell viability incubated with DNA Gel,

DOX@DNA Gel, DOX@DNA Gel + ATP (400 μM) was tested in a similar way.

Cellular Uptake Assays. A Calibur flow cytometer (Becton Dickinson, Franklin Lakes, NJ) was used to monitor the fluorescence of DOX within the cells. B16 cells were seeded in a 12-well plate at a density of 1 × 10^5 cells per well in 1 mL of RPMI-1640 medium. After overnight incubation, the medium of each well was replaced with 1 mL of fresh medium containing DOX@DNA Gel (100 μL) with different ATP concentrations (4, 10, 40, and 400 μM) for 8 h. After that, the culture medium was discarded, and the cells were washed with PBS for three times, digested, centrifuged, and resuspended in PBS (1 mL) before flow cytometry analysis. Cells treated with PBS were used as control. For each sample, 1 × 10^4 cells were counted and the measurement was repeated for 3 times.

The confocal laser scanning microscopy (CLSM, Carl Zeiss LSM 700, Jena, Germany) was further used to qualitatively confirm the intracellular uptake. In brief, B16 cells were seeded in confocal dishes at a density of 2 × 10^5 cells per dish with 1.0 mL medium and incubated overnight. After that, the medium in each dish was substituted with fresh medium containing free DOX, DOX@DNA Gel, or aPDL1/DOX@DNA Gel at a DOX concentration of 2.5 μg/mL for 2 or 8 h. Whereafter, the culture medium was removed and the cells were washed with PBS for three times. Then, the cells were fixed with glutaraldehyde (2.5%) for 15 min and counter stained with DAPI for 15 min at 37 °C for CLSM observation.

***In Vitro* Immunogenic Cell Death.** To verify the immunogenic cell death (ICD) effect of cancer cells, CRT expression on B16 cells was investigated by flow cytometry. In brief, B16 cells were treated with DNA Gel (DNA 0.7 μg/mL), DOX@DNA Gel (DNA 0.7 μg/mL, DOX 5 μg/mL) or free DOX (5 μg/mL) for 8 h. Then the cells were washed and incubated with Alexa Fluor® 488 Anti-Calreticulin (CRT) antibody for 0.5 h before analysized by flow cytometer. Cells with PBS treatment was used as control. For each sample, 1 × 10^4 cells were counted and the measurement was repeated for 3 times.

***In Vitro* Maturation of Dendritic Cells.** DCs were cultured in DMEM supplemented with 10% FBS and 1% penicillin-streptomycin at 5% CO_2 and 37 °C. Then, DCs were mixed with DNA Gel (10 μg, 0.1 mL) or CpG oligodeoxynucleotide (ODN) (2.1 μg, 0.1 mL) and incubated for 24 hours. After that,

DC cells were collected and incubated with CD80 and CD86 antibodies. Three parallel experiments were performed in each group. The sequences of CpG OND (1826) are as follows: 5'-TCC ATG ACG TTC CTG ACG TT-3'. The cell supernatants were also collected for an enzyme-linked immunosorbent assay (ELISA) to quantify cytokines IL-12, and IL-6 expressed by DCs.

A 12-well transwell system with 0.4-μm polycarbonate porous membranes was used to analyze the ICD-induced maturation of DCs. In brief, B16 cells were seeded in the upper wells at a density of 1×10^5 cells per well with 1 mL of medium and incubated overnight. Then, the cells were treated with 100 μL DNA Gel (DNA = 0.7 μg/mL), DOX@DNA Gel (DNA = 0.7 μg/mL, DOX = 5 μg/mL) or free DOX (5 μg/mL) for 24 h. B16 cells treated with normal saline (NS) were used as control. Meanwhile, DCs were seeded in the lower wells at a density of 1×10^5 cells per well with 1 mL of medium and incubated overnight. Subsequently, the upper wells were merged with the lower wells for mixed culture of both B16 cells and DCs. After 24 h, DCs were collected and stained with anti-CD86 or anti-CD40 antibody for 15 min in the dark before flow cytometry analysis. The culture medium was collected to detect the secretion of IL-6 and IL-12 by DCs.

***In Vivo* Biocompatibility.** The ability of immunoadjuvant to induce splenomegaly was evaluated. Mice were housed under normal conditions with 12 h light and dark cycles at 25 °C, 40% relative humidity with free access to food and water.

Particularly, healthy C57BL/6 mice (15-20 g, 3-4 weeks, female, Shanghai Slac Laboratory Animal Center, Shanghai, China) were randomly divided into 3 groups (n = 3 in each group), and subcutaneously injected with 100 μL of NS, CpG ODN, or DNA Gel, respectively, on day 0. Then, the heart, liver, lung, spleen, and kidney of mice were harvested after they were anesthetized on day 3. The spleens were photographed and weighted, and the organ samples were processed, sectioned, and hematoxylin and eosin (H&E) stained for further observation of the histological changes. The blood samples were also collected on day 3 after the immunization and were centrifugated at 2000 rpm for 3 min to obtain the serum. The serum biochemistry markers including alanine

aminotransferase (ALT), aspartate aminotransferase (AST), creatinine (CREA), and blood urea nitrogen (BUN) were analyzed by Servicebio Technology Co., Ltd (Wuhan, China).

***In Vivo* Anti-Tumor Therapeutic Efficacy.** To establish a xenografted tumor model, female C57BL/6 mice (15-20 g, 3-4 weeks) were subcutaneously injected with 1×10^6 B16 cells (in 100 μL of NS, for each mouse) in the right back leg. When the tumor volume reached about 100 mm^3, B16 tumor-bearing C57BL/6 mice were randomly divided into 6 groups (5 mice for each group). Since the prevalent intratumoral administration of hydrogels has attracted much attention in both fundamental research and preclinical setting, the NS, DNA Gel, aPDL1@DNA Gel, free DOX, DOX@DNA Gel, and aPDL1/DOX@DNA Gel were intratumorally administrated (100 μL, DOX dose = 72.5 μg, aPDL1 dose = 90 μg, and DNA dose = 10 μg) into mice every 3 days for 2 consecutive times. Tumor volume was measured at 2 days interval and calculated as follows:

$$V = W^2 \times L/2 \quad (4)$$

W and L represented the length in minor and major axes, respectively. Relative tumor volume was calculated as V/V_0 (V_0, tumor volume of mice on the first day; V, tumor volume after treatment at a given time point).

Histological Examinations of Tumor Tissues. For histological analysis, the tumor-bearing mice were sacrificed at day 14, and part of tumor tissues were excised, fixed in 4% paraformaldehyde overnight, embedded in paraffin and sectioned into slices for CRT staining, hematoxylin and eosin (H&E) staining and TdT-mediated dUTP Nick-End Labeling (TUNEL) staining. The percentage of CRT or TUNEL positive cells in each group is quantified. The circular chart visually compares the percentage of TUNEL positive cells in each group.

***In Vivo* Analysis of Immune Activation**. To verify vaccination-induced immune response, serum samples were collected from treated mice (n = 3). Samples were diluted and the serum concentrations of IL-6 and IL-12 were analyzed by the corresponding ELISA kits.

Harvested tumor tissue (n = 3) were cut into small pieces and incubated with a digestion solution (1 mg/mL type IV collagenase, 100 μg/mL DNase I and 100 μg/mL hyaluronidase in RPMI-1640

medium) at 37 °C for 30 min with persistent agitation. Then, the digested tumors were ground in a 200-mesh sieve to obtain a single cell suspension of tumor-infiltrating tissue, followed by centrifugation (1000 rpm, 5 min) and re-suspension in DMEM. Afterwards, the lymphocytes in the single cell suspension of tumor-infiltrating tissue were separated according to the instructions of the tumor infiltrating lymphocyte cell separation medium kit (mice). In brief, the prepared single cell suspension of tumor-infiltrating tissue was mixed with equal volume of lymphocytes separation solution, followed by centrifugation (500 g, 30 min) at room temperature. After centrifugation, the second lymphocyte layer was carefully separated, washed with cell washing solution, and collected to obtain tumor-infiltrating lymphocytes. Then, the lymphocytes were resuspended in DMEM and filtered by the nylon wool column to extract tumor-infiltrating T cells.

For the analysis of tumor-infiltrated T cells, the extracted T cells were resuspended in PBS and stained with anti-CD3, anti-CD4 and anti-CD8 (5 μL of each) for 15 min in the dark, washed with PBS, and resuspended in PBS before flow cytometry analysis (n = 3).

***In Vivo* Evaluation of Anti-Metastasis Effect.** A B16 bloodstream metastasis model was established to evaluate the anti-metastasis effect of aPDL1/DOX@DNA Gel. In brief, the B16 tumor-bearing mice with a tumor volume of about 100 mm^3 were randomly assigned to 3 groups (n = 8 in each group) and intravenously administrated with 0.1 mL NS, or 0.1 mL NS containing aPDL1+DOX+CpG (DOX dose = 72.5 μg, aPDL1 dose = 90 μg, and DNA dose = 10 μg), or aPDL1/DOX@DNA Gel (100 μL, DOX dose = 72.5 μg, aPDL1 dose = 90 μg, and DNA dose = 10 μg) every 3 days for 3 times, respectively. On day 14, 1×10^6 B16 cells were administered intravenously *via* tail vein infusion into each C57BL/6 mouse. The tumor volume and body weight of each mouse were recorded every other day for 14 days, and the survival rate of mouse in all groups (n=5) was measured. The survival rate of the mice in each group was calculated following the reported formula:

$$N_1/N \times 100\% \qquad (5)$$

where N_1 and N stand for the number of survival mice and total mice in each group, respectively.

After one week, the tumor-bearing mice were sacrificed, and the heart, liver, spleen, lung, kidney

and tumor tissues were excised, fixed in 4% paraformaldehyde overnight, and embedded in paraffin for hematoxylin and eosin (H&E) staining to observe the histological changes. The pulmonary metastatic nodules were examined by H&E staining lung sections, and tumors were also sectioned into slices for TUNEL staining, which were applied to detect the apoptosis rate of tumor cells.

***In Vivo* Analysis of Immune Memory Cells and Cytokines.** To investigate the immunological effect induced by different treatments, the spleens were collected under sterile conditions and stored in icy PBS solutions. To extract T cells in spleens, the spleens were cut into small pieces, ground in a 200-mesh sieve, suspended in DMEM, followed by centrifugation (1000 rpm for 5 min) and re-suspension in DMEM. Then, the single cell suspension was passed through a nylon wool column to obtain the splenic T cells. For the analysis of memory T cells, cell suspension harvested from spleens were stained with anti-CD8, anti-CD44 and anti-CD62L to confirm their phenotype populations.

Meanwhile, the serum of the tumor-bearing mice was collected and isolated to analyze the expression levels of immune-related cytokines including interferon-γ (IFN-γ), IL-6, and TNF-α by ELISA. Other serum biochemistry markers including ALT, AST, CREA, and BUN were also analyzed.

Biodistribution of DNA Gel. B16 tumor-bearing mice were intratumorally administered with the synthesized Cy3-labeled DNA Gel (10 μg DNA). After 6 h, main organs including heart, liver, spleen, lung, kidney, and tumor were extracted and then imaged on a commercial VISQUE *In Vivo* Smart Imaging System. Cy3-labeled CpG ODN was used as control. For fluorescent labeling of DNA Gel, Cy3-dUTP (0.02 mM) was added into reaction mixtures during RCA synthesis.

Statistical Analysis. All experimental data were represented as mean ± SD with at least three parallel samples or measurement. Statistical analyses were conducted using GraphPad Prism 7. Unpaired two-tailed Student's t test or one-way analysis of variance (ANOVA) was used for data analyses. A value of 0.05 was set as the significance level, and the data were marked with (*) $p < 0.05$, (**) $p < 0.01$, and (***) $p < 0.001$, respectively.

Table S1. List of DNA sequences.

Name	Sequence (5'-3')
Template (ATP aptamer complementary sequences and CpG complementary sequences)	GCAATACTCCCCCAGGTGTTAGATGCTGCTGCAGCGATA CGCGTATCGCTATTCCATGACGTTCCTGACGTTGCAGCAG CCACCTGGAACCTTCCTCC
Primer (ATP aptamer)	CACCTGGGGGAGTATTGCGGAGGAAGGTTCCAGGTG
Template-1	TTCCCGGCGGCGCAGCAGTTAGATGCTGCTGCAGCGAT ACGCGTATCGCTATGGGTAACCGTACGGTTACCCGCAG CAGCATCTAACCGTACAGTA TT
Primer-1	TAACTGCTGCGCCGCCGGGAAAATACTGTACGGTTA

Table S2. The drug loading efficiency (DLE) and drug content (DLC) of DOX@DNA Gel.

Volume of DOX solution (2 mg/mL) /μL	Drug content /μg	DOX loading efficiency (DLE)	Drug loading content (DLC)
2.5	4.99	99.80 %	48.85 %
5	9.82	98.20 %	65.27 %
10	19.45	97.25 %	78.82 %
20	35.69	89.23 %	87.23 %
50	40.51	40.51 %	88.58 %

Table S3. Quantification of different sample (in 50 μL volume).

Sample	DNA content (μg)	DOX content (μg)	aPDL1 content (μg)
DNA Gel	5.06±0.31	-	-
aPDL1@DNA Gel	4.84±0.15	-	45.02
DOX@DNA Gel	5.06±0.31	36.25±1.63	-

aPDL1/DOX@DNA Gel	4.84±0.15	35.65±3.23	46.40

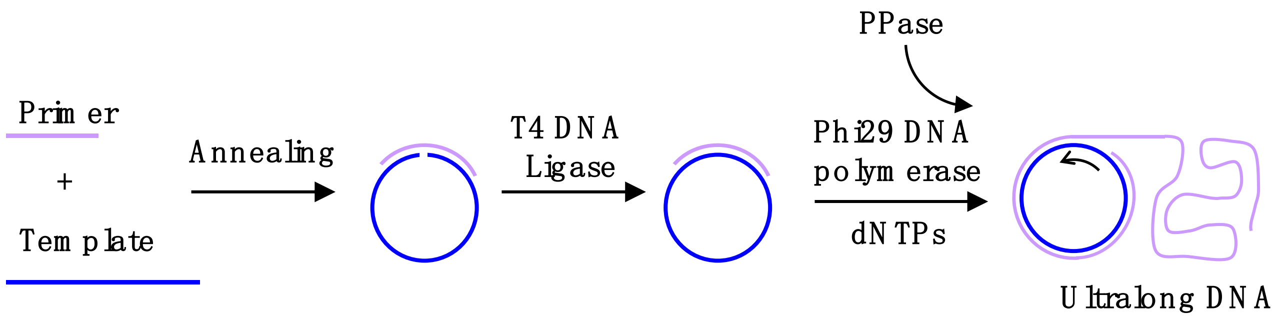

Figure S1. Schematics of synthesis of ultralong DNA chain *via* RCA reaction.

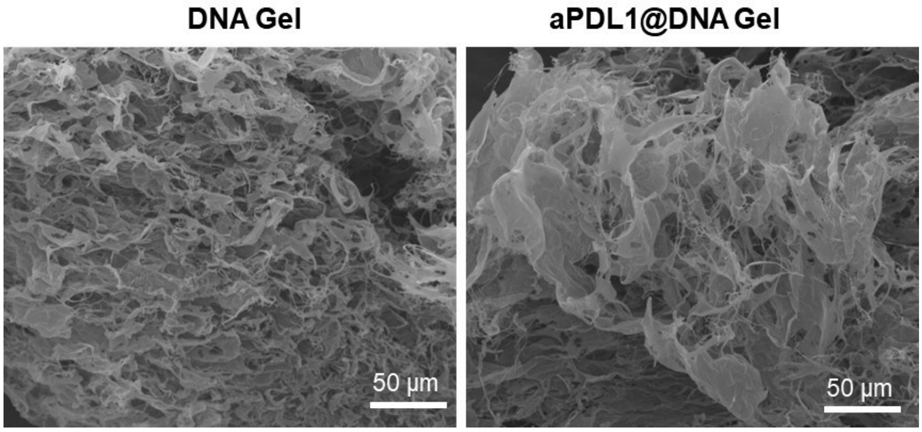

Figure S2. SEM images of DNA Gel and aPDL1@DNA Gel after lyophilization, respectively.

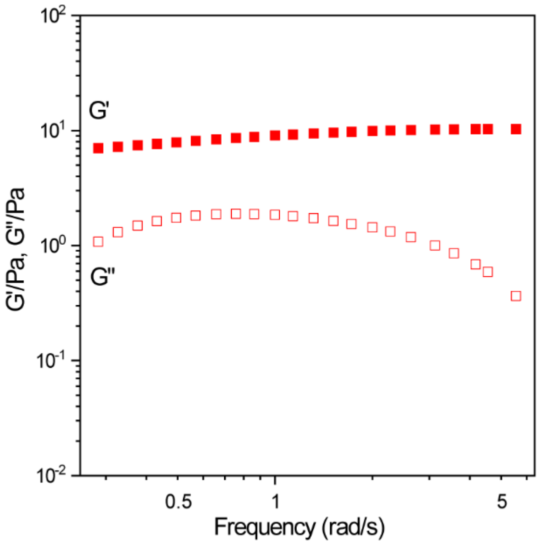

Figure S3. Storage (G′) and loss (G″) modulus of DNA Gel as a function of frequency. Storage modulus value (G′) is constantly higher over shear-loss modulus value (G″) in the entire frequency range, confirming the formation of hydrogel.

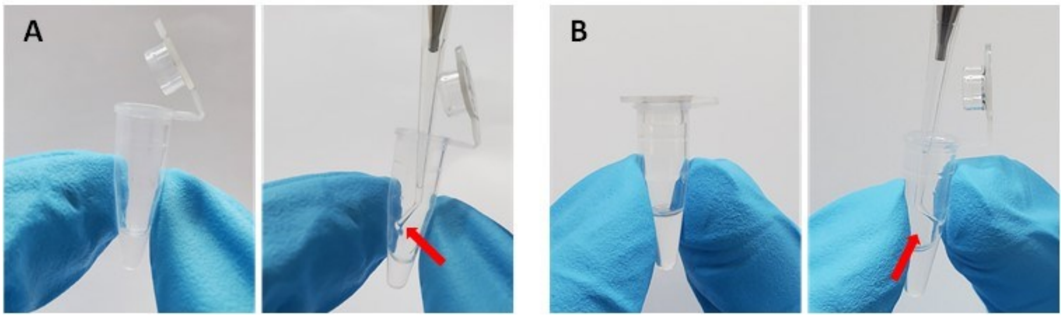

Figure S4. Photographs of (A) DNA Gel and (B) aPDL1@DNA Gel. The red arrows point at elastic hydrogel filaments that can be stretched like a rubber band.

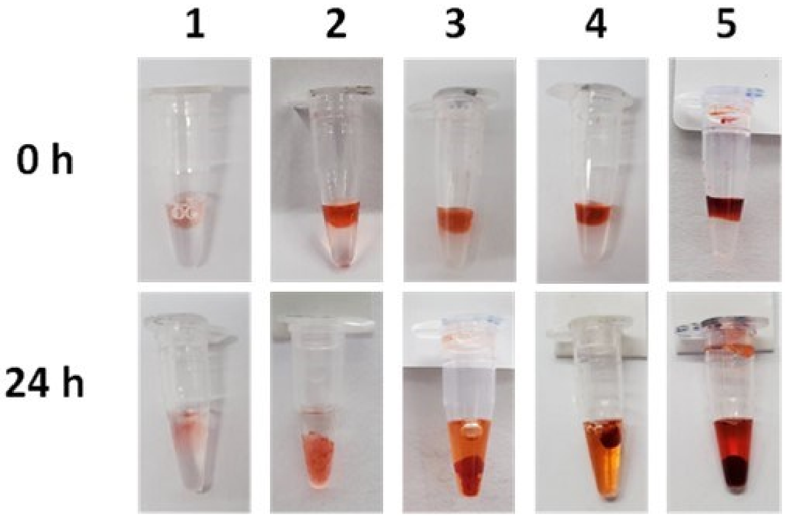

Figure S5. Photographs of DNA Gels before (0 h) and after DOX loading (24 h). 1: 50 μL DNA Gel + 47.5 μL H_2O + 2.5 μL DOX solution (2 mg/mL); 2: 50 μL DNA Gel + 45 μL H_2O + 5 μL DOX solution (2 mg/mL); 3: 50 μL DNA Gel + 40 μL H_2O + 10 μL DOX solution (2 mg/mL); 4: 50 μL DNA Gel + 30 μL H_2O + 20 μL DOX solution (2 mg/mL); 5: 50 μL DNA Gel + 50 μL DOX solution (2 mg/mL).

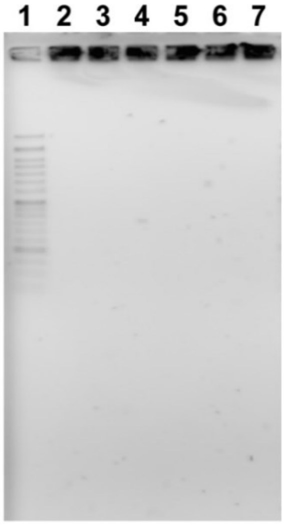

Figure S6. Agarose gel electrophoresis (2%) analysis of the storability of aPDL1/DOX@DNA Gel in normal saline at 4 °C for 0 (lane 2), 1 (lane 3), 2 (lane 4), 3 (lane 5), 4 (lane 6), and 5 d (lane 7), respectively. Lane 1: 50 bp DNA marker.

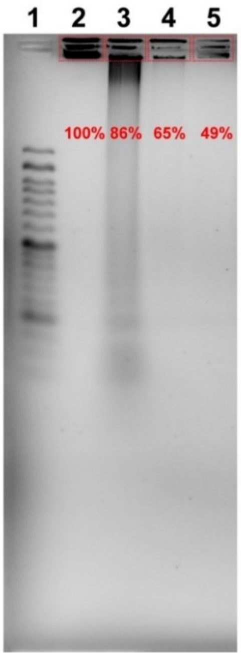

Figure S7. Agarose gel electrophoresis (2%) analysis of aPDL1/DOX@DNA Gel after incubation with DNase I (5 U/mL) at 37 °C for 0 (lane 2), 2 (lane 3), 6 (lane 4), and 12 h (lane 5), respectively. The relative amount of hydrogel in the pocket (red dotted box) was semi-quantified through analysis of gray value of gel band using ImageJ. Lane 1: 50 bp DNA marker. Of note, unlike DNA oligonucleotide that can be completely degraded within a few minutes, aPDL1/DOX@DNA Gel still maintained approximately 50% integrity even after 12 h incubation with 5 U/mL DNase I.

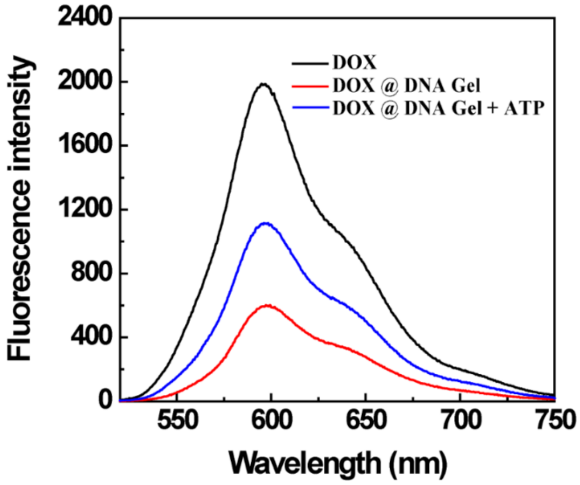

Figure S8. Fluorescence spectra of DOX, DOX@DNA Gel, and DOX@DNA Gel + ATP (400 μM), respectively. Of note, the recovery of fluorescence intensity in DOX@DNA Gel + ATP is attributed to the DOX being released from the conformationally changed aptamer, which eliminates the donor-quencher FRET between DOX and aptamer.

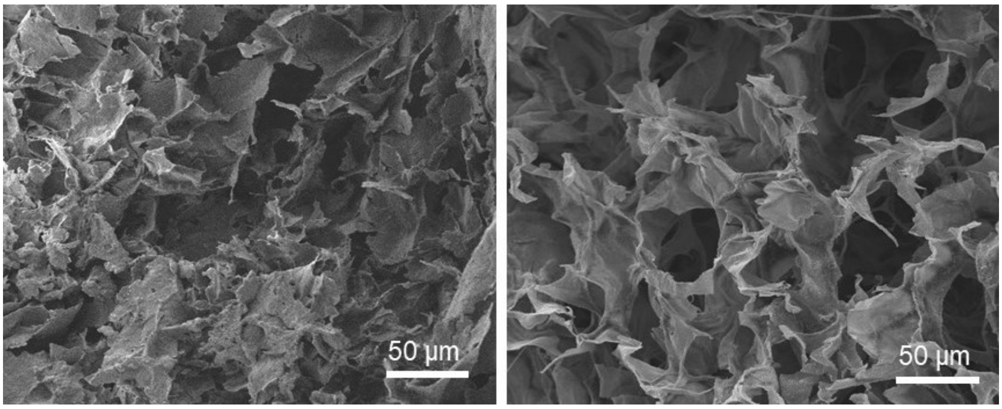

Figure S9. SEM images of aPDL1/DOX@DNA Gel before (left) and after (right) incubation with ATP (400 μM) for 5 h.

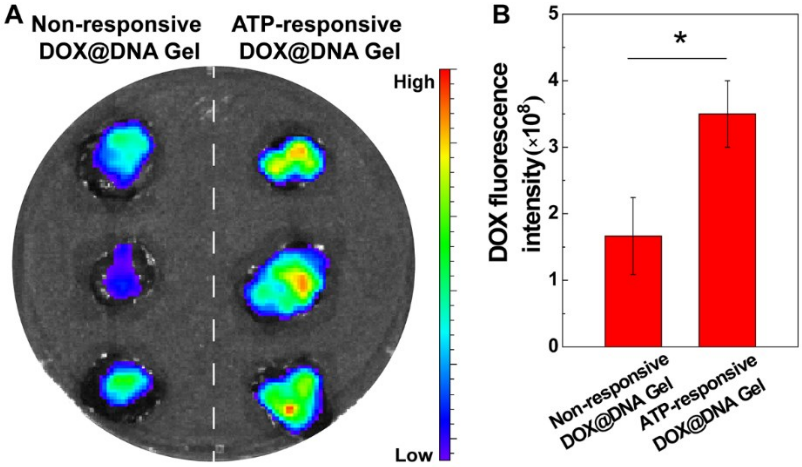

Figure S10. (A) Ex vivo fluorescence imaging of tumor tissues treated with ATP-responsive DNA Gel and non-responsive DOX@DNA Gel, respectively. (B) Statistical analysis of fluorescence intensity according to fluorescence images. Of note, ATP-responsive DOX@DNA Gel displayed the stronger DOX fluorescence intensity than non-responsive DOX@DNA Gel in tumor sites, which is attributed to the weakened donor-quencher FRET between DOX and aptamer in the presence of ATP. Unless otherwise stated, ATP-responsive DNA Gel was used.

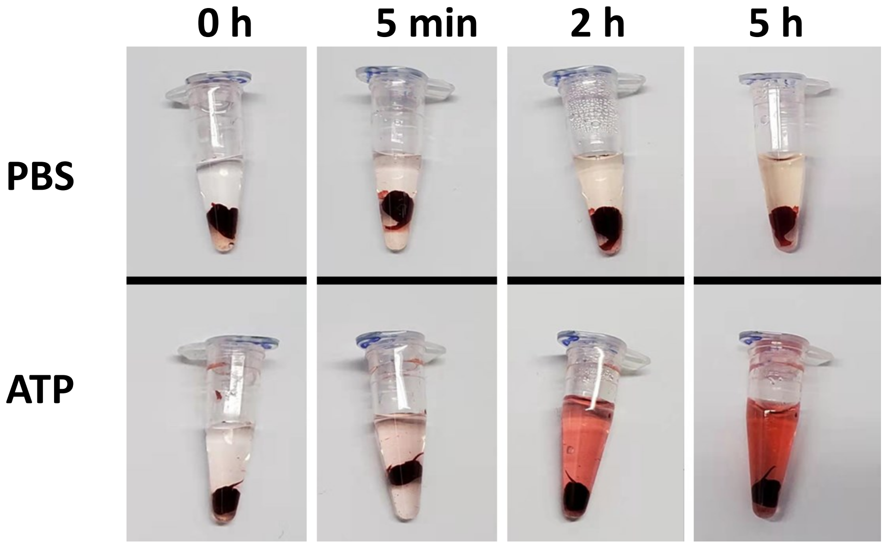

Figure S11. Photographs of aPDL1/DOX@DNA Gel incubated in PBS buffer with/without ATP (400 μM) at different times.

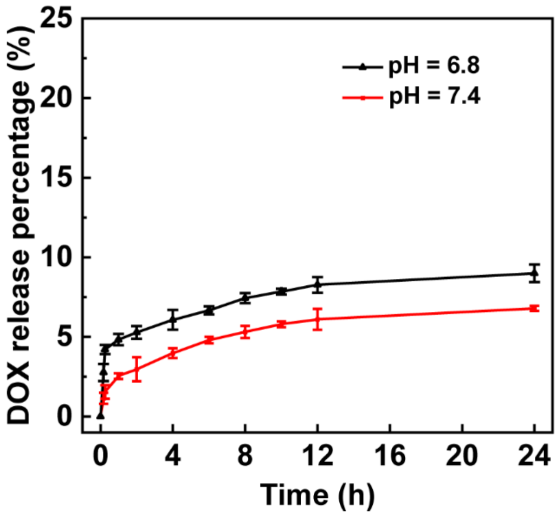

Figure S12. Cumulative DOX release curves of DOX@DNA Gel at pH 6.8 and 7.4.

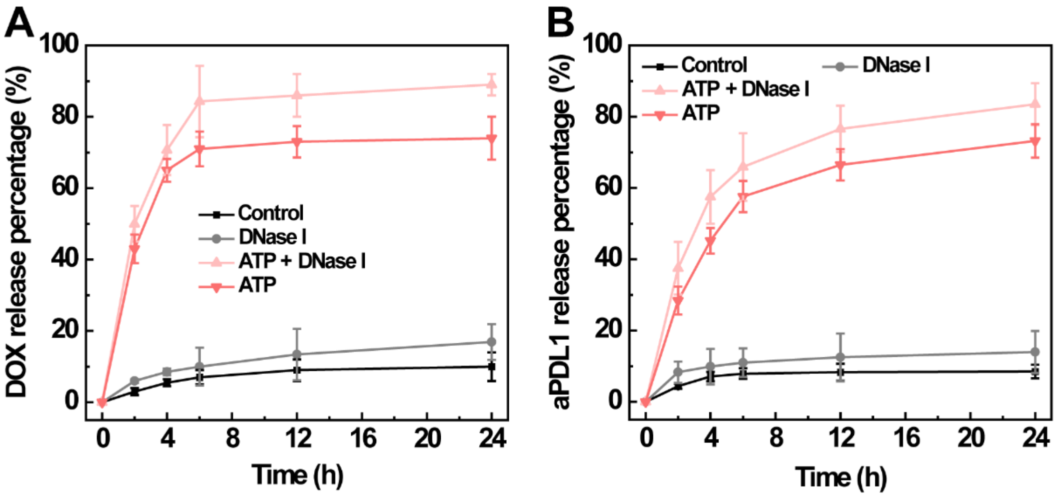

Figure S13. Release profiles of (A) DOX and (B) aPDL1 from DNA Gel in the presence of ATP (4 mM) and/or DNase I (1 U/mL).

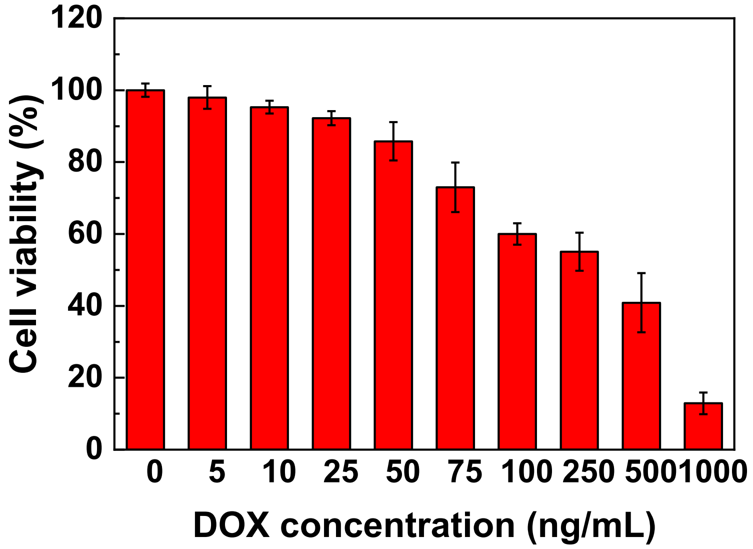

Figure S14. Viability of B16 cells after treated with free DOX at different concentrations for 24 h.

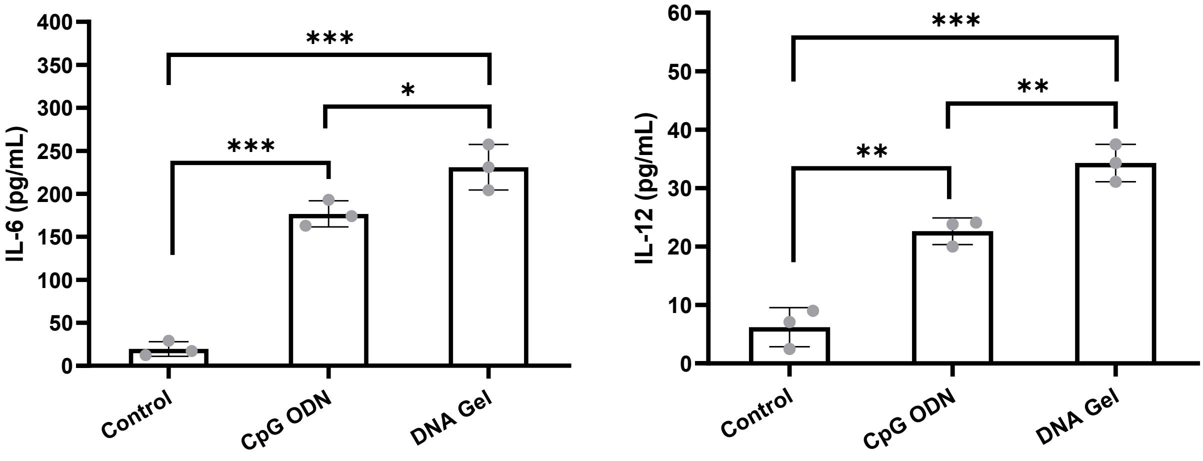

Figure S15. IL-6 and IL-12 secreted by mature DCs after 24 h incubation with different materials (n = 3 for each sample, and *, **, and *** represent $p < 0.05$, $p < 0.01$, and $p < 0.001$, respectively).

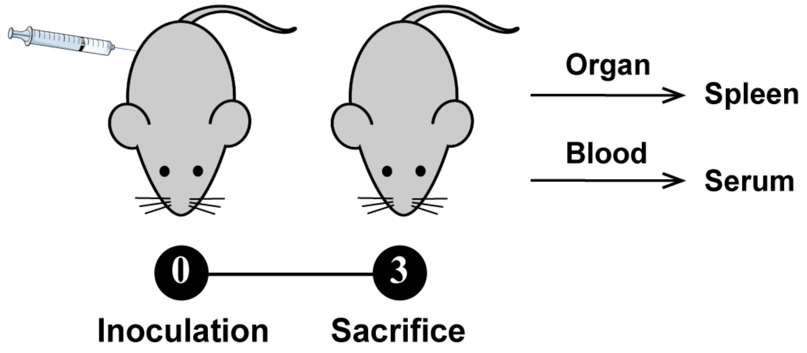

Figure S16. Schematic illustration of the vaccination via subcutaneous injection of DNA Gel or free CpG ODN.

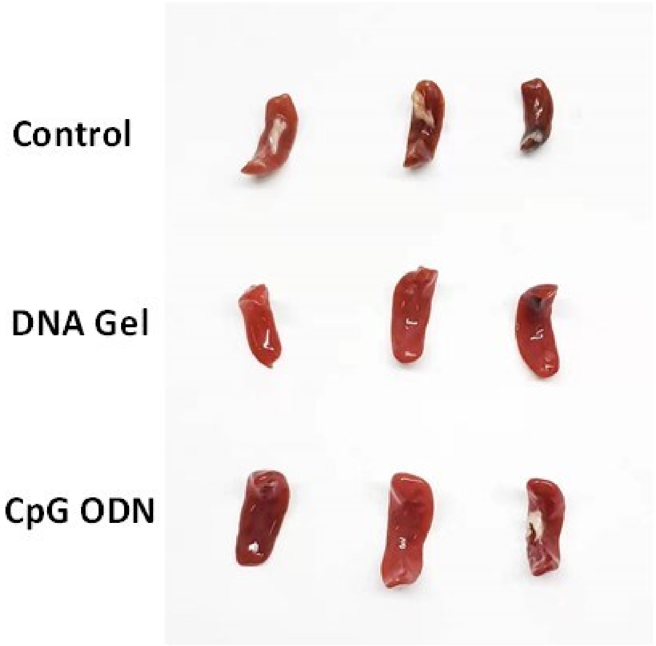

Figure S17. Representative images of spleens from mice subcutaneously injected with PBS, DNA Gel, and free CpG ODN, respectively.

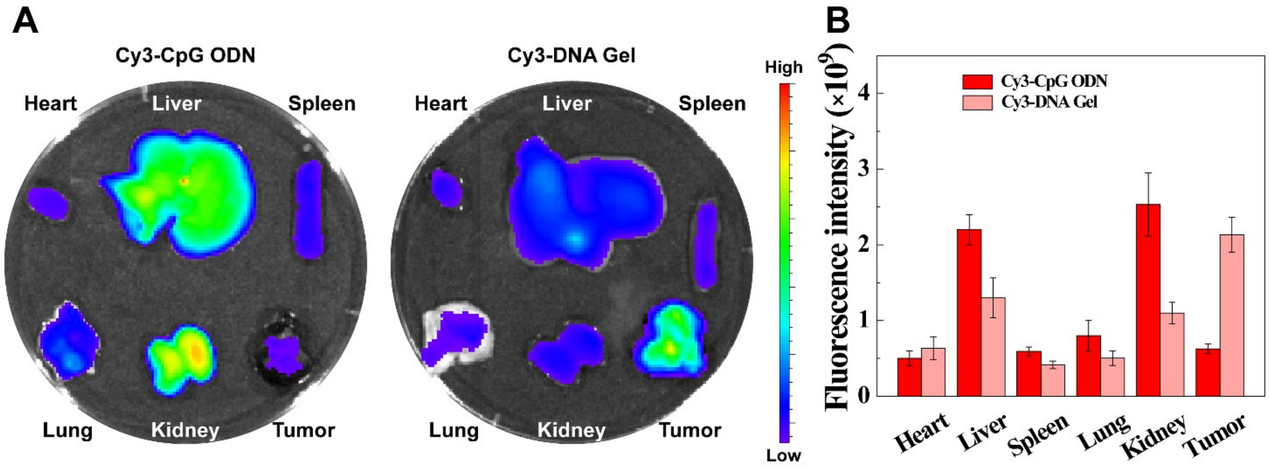

Figure S18. (A) *Ex vivo* fluorescence imaging of heart, liver, spleen, lung, kidney, and tumor at 6 h post-injection of Cy3-labeled CpG ODN and Cy3-labeled DNA Gel, respectively. (B) Statistical analysis of fluorescence intensity according to fluorescence images.

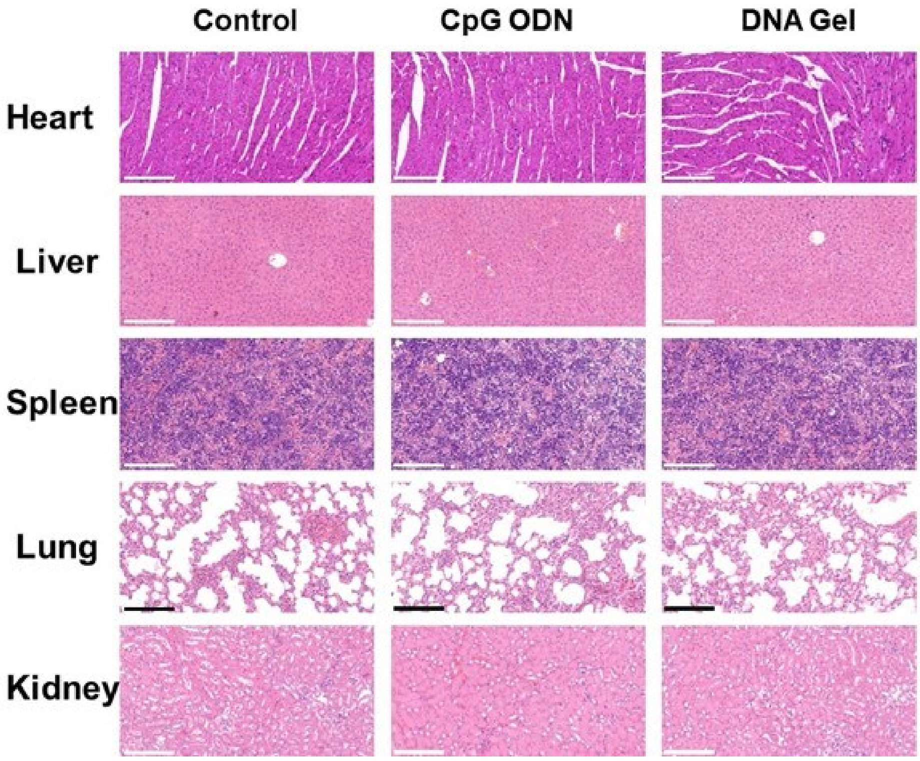

Figure S19. H&E staining of slices of the heart, liver, spleen, lung and kidney. The scale bar in each panel represents 100 μm.

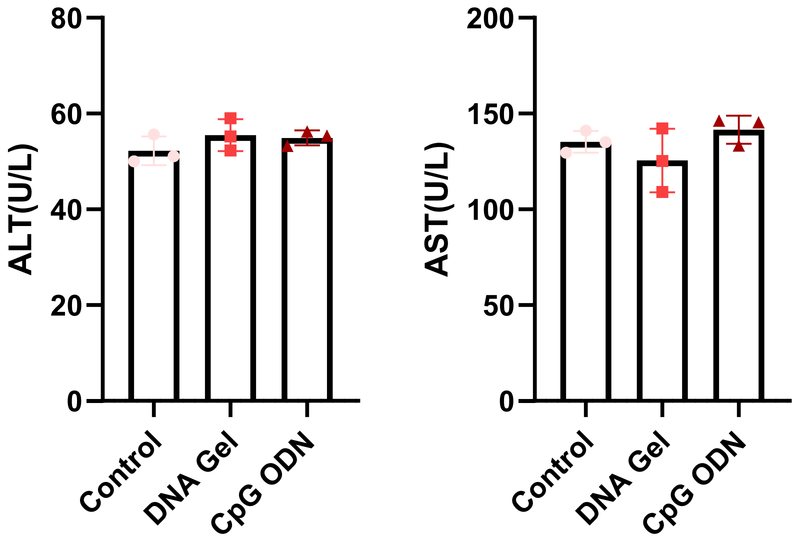

Figure S20. The liver function markers including alanine aminotransferase (ALT) and aspartate aminotransferase (AST) of mice at the end of treatments. Data was shown as mean ± SD (n = 3).

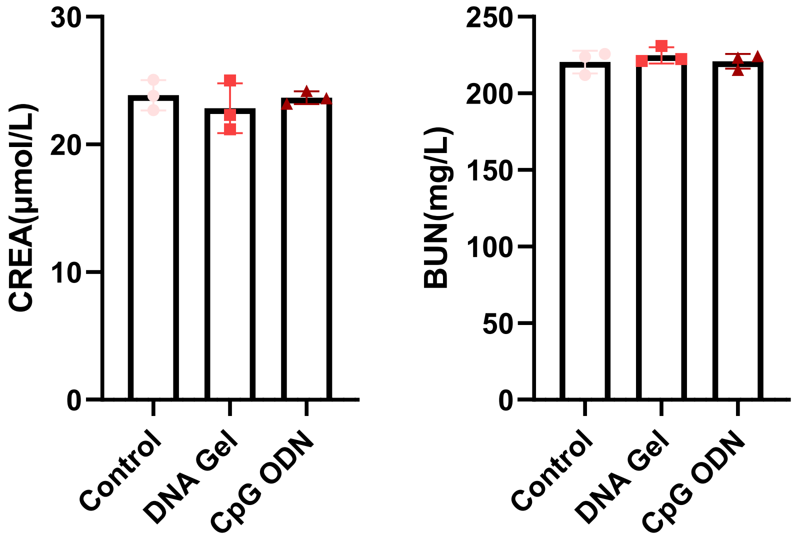

Figure S21. The kidney function marker creatinine (CREA) and blood urea nitrogen (BUN) of mice at the end of treatments. Data was shown as mean ± SD (n = 3).

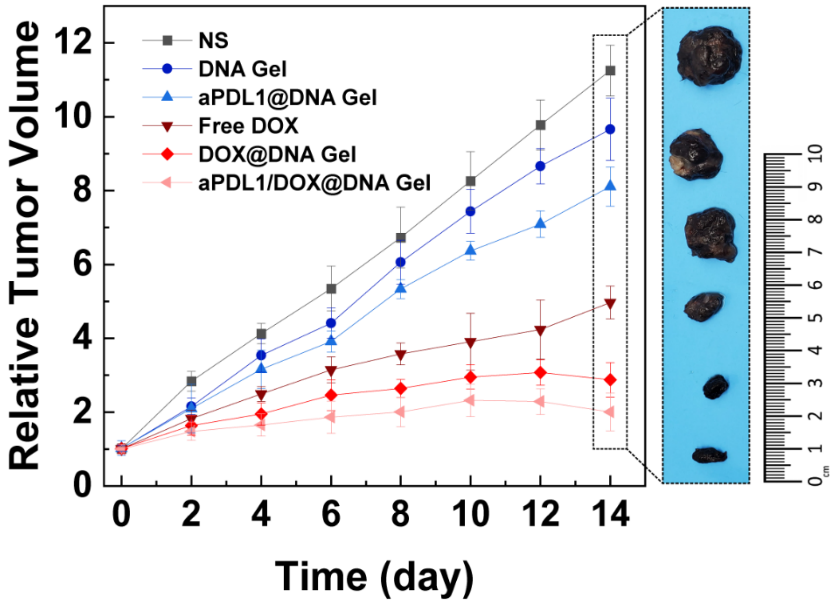

Figure S22. Average tumor growth curves of mice after different treatments. The insets depict representative photographs of the corresponding tumors.

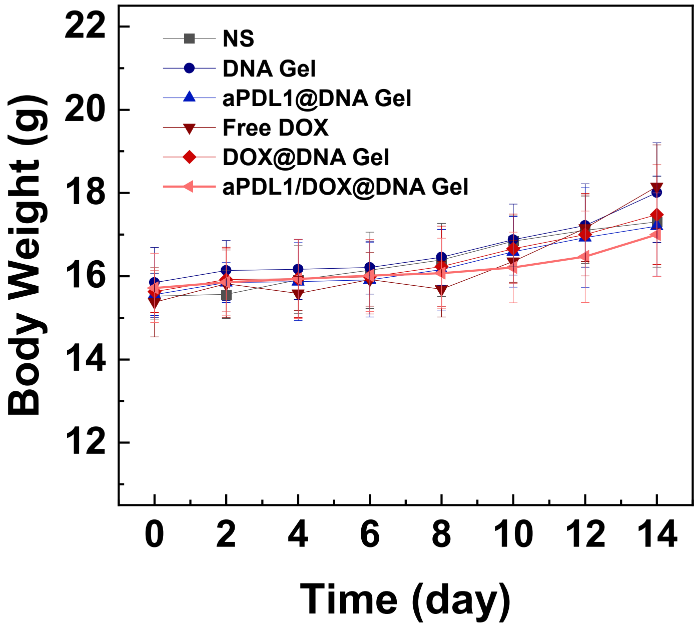

Figure S23. Body weights of mice after different treatments. No obvious decrease in body weight was observed for all the treatments.

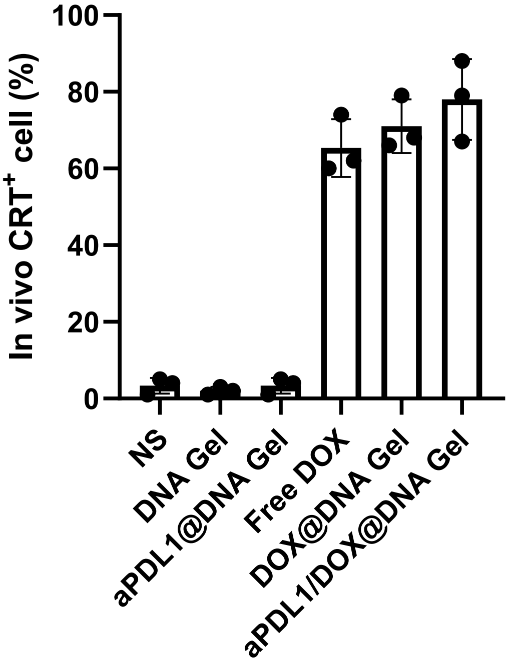

Figure S24. Quantitative analysis of CRT-positive cells in tumor sections after various treatments.

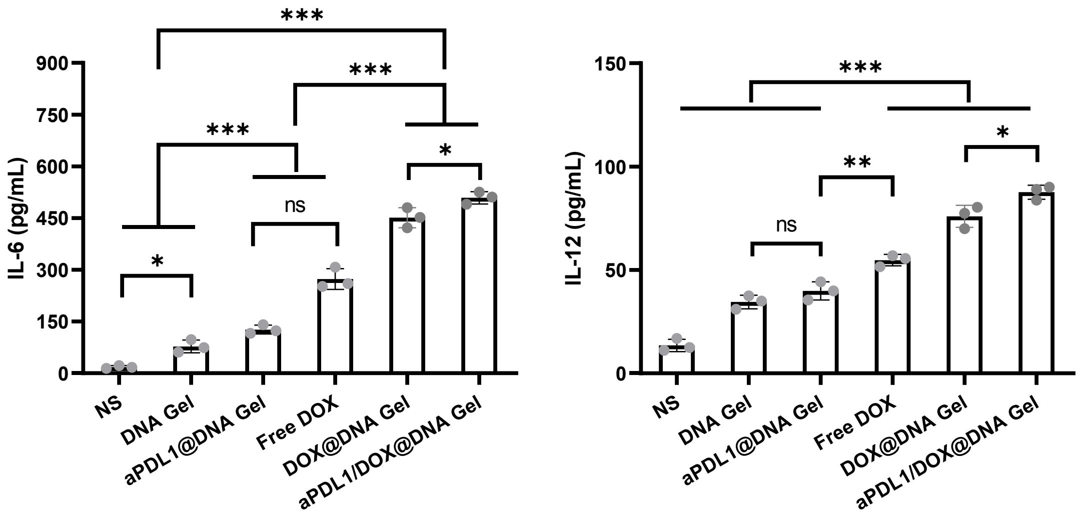

Figure S25. The levels of IL-6 and IL-12 in serum of mice after various treatments for 14 days (n = 3, ns means not significant, *** means p < 0.001, ** means p < 0.01, * means p < 0.05).

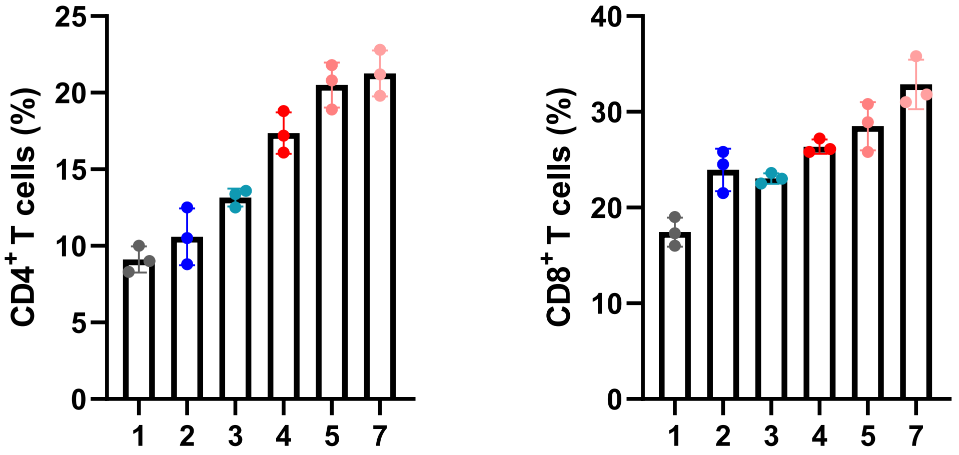

Figure S26. The percentage of CD4+ and CD8+ T cells in total immune cells within the tumor after different treatments (n = 3). Data are represented as mean ± SD. 1: NS; 2: DNA Gel; 3: aPDL1@DNA Gel; 4: free DOX solution; 5: DOX@DNA Gel; 7: aPDL1/DOX@DNA Gel.

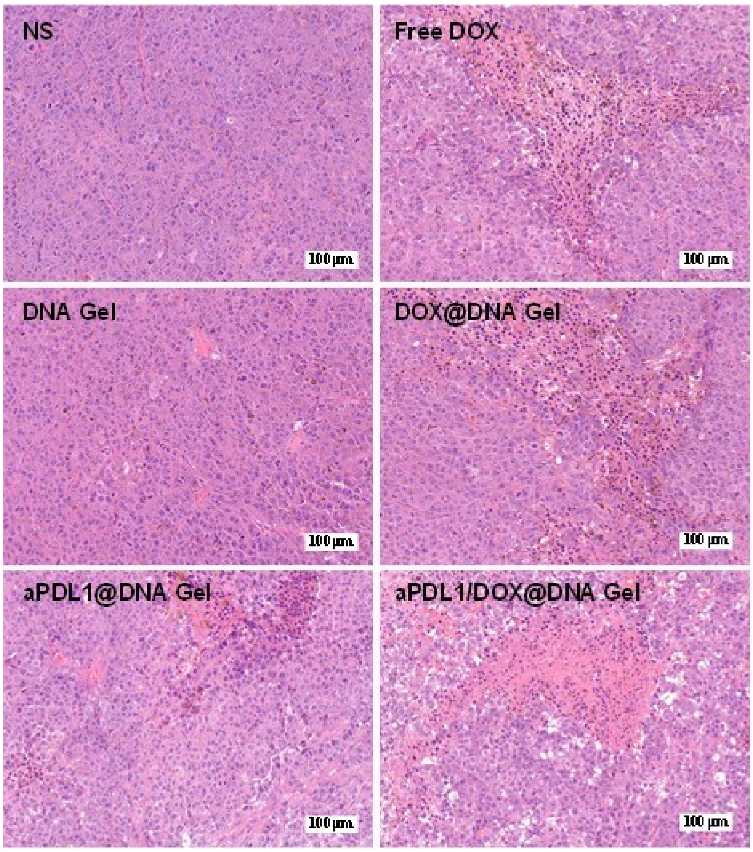

Figure S27. H&E staining of tumor tissues collected from the tumor-bearing mice after different treatments.

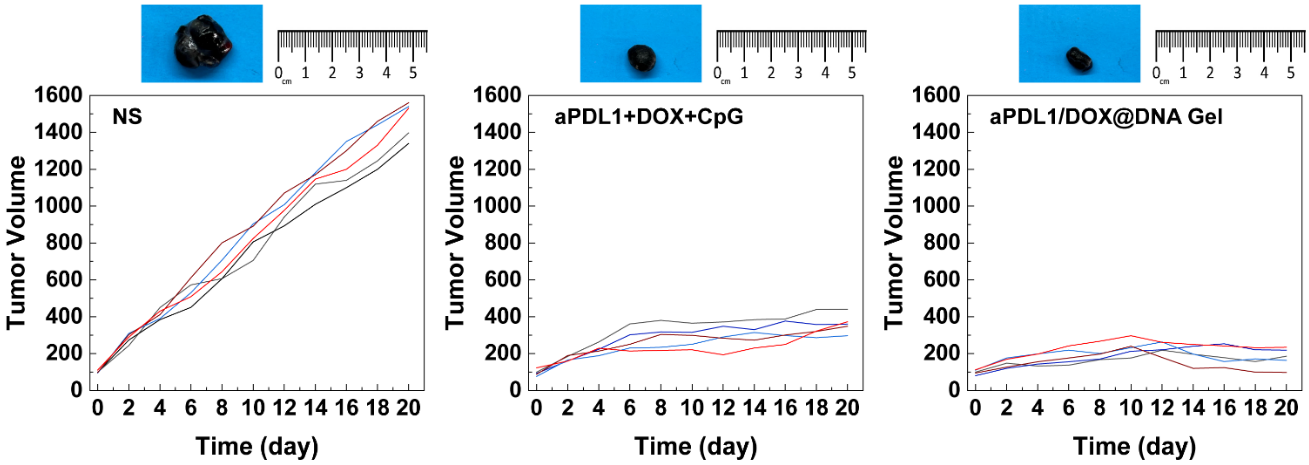

Figure S28. Individual tumor growth profiles of mice after different treatments. The insets depict representative photographs of the corresponding tumors.

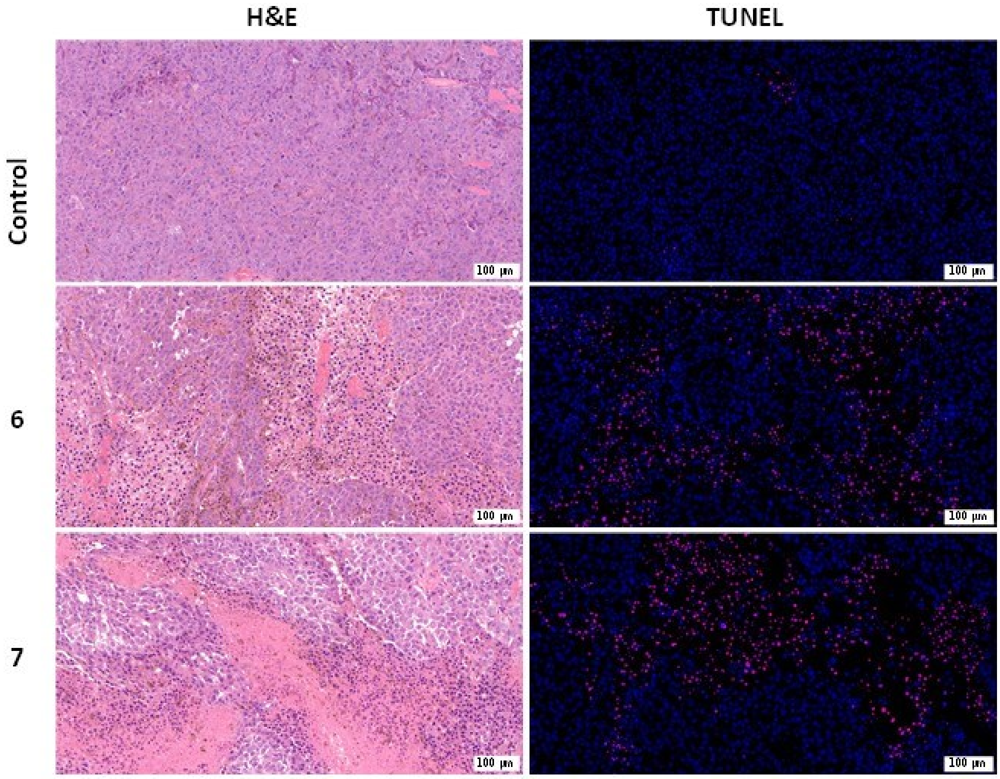

Figure S29. H&E and TUNEL staining of tumor tissue sections collected from the tumor-bearing mice after different treatments. 6: aPDL1+DOX+CpG; 7: aPDL1/DOX@DNA Gel.

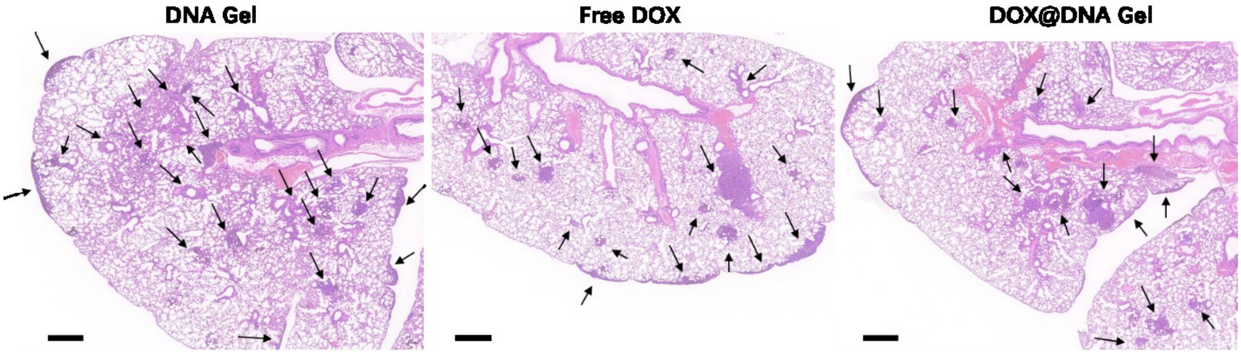

Figure S30. Histological images of lung metastatic foci of mice after different treatments. Metastatic foci are indicated with black arrow. Scale bars: 500 μm.

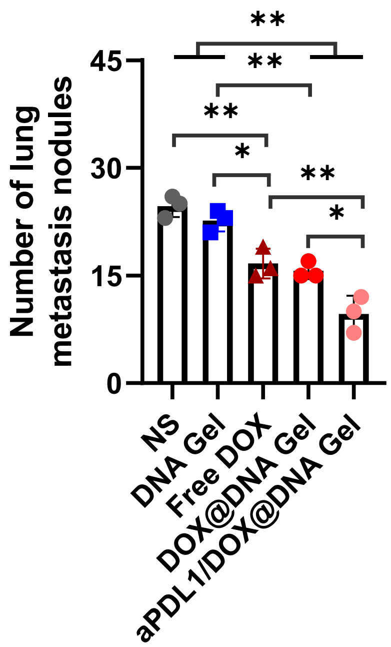

Figure S31. Statistical analysis of lung metastatic foci of mice after treatments with NS, DNA Gel, free DOX, DOX@DNA Gel, and aPDL1/DOX@DNA Gel, respectively, according to histological images shown in Figures 5F and S30. Of note, the capability for inhibiting lung metastatic foci follows the order of aPDL1/DOX@DNA Gel > DOX@DNA Gel > free DOX > DNA Gel > NS. Clearly, DOX synergizing with CpG ODN in DNA Gel plays a pivotal role in anti-metastatic effect, while anti-metastatic effect can be further enhanced by the inclusion of aPDL1.

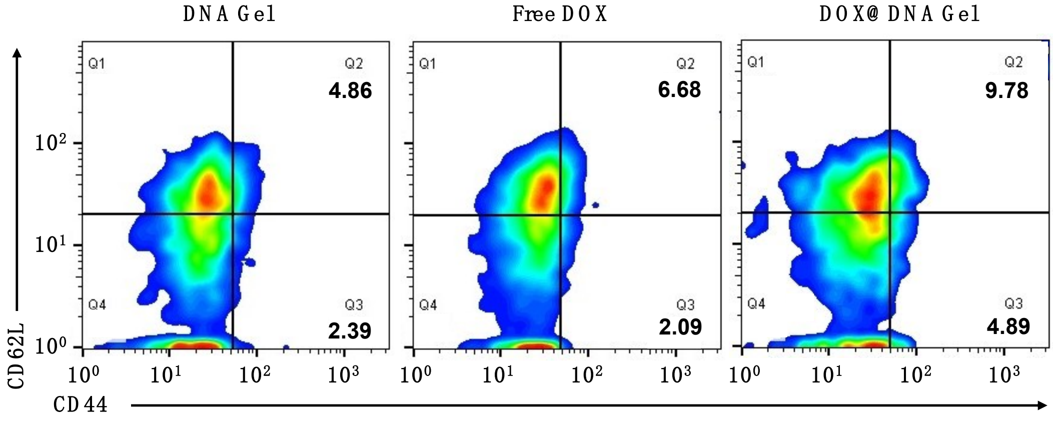

Figure S32. Flow cytometry analysis of the proportions of T_{CM} cells (CD44+CD62L+ T cells gated on CD8+ T cells) and T_{EM} cells (CD44+CD62L- T cells gated on CD8+ T cells) in spleens of mice after treatment with DNA Gel, free DOX, and DOX@DNA Gel, respectively.

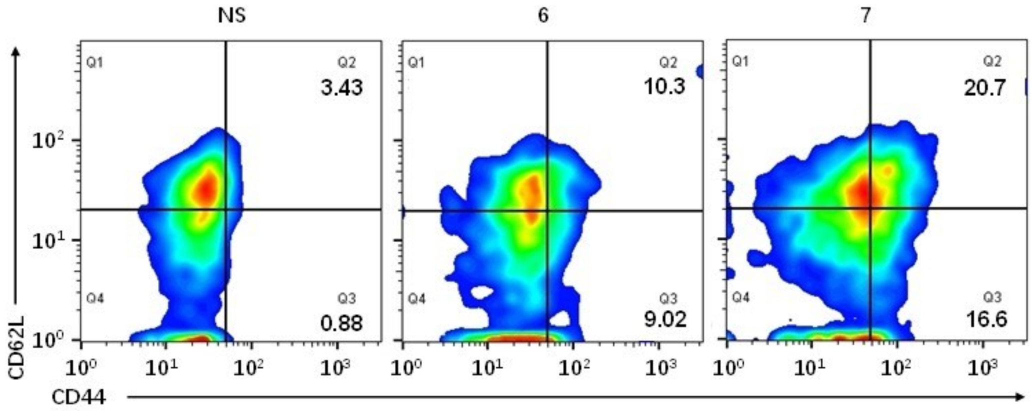

Figure S33. Flow cytometry analysis of the proportions of T_{CM} cells (CD44+CD62L+ T cells gated on CD8+ T cells) and T_{EM} cells (CD44+CD62L- T cells gated on CD8+ T cells) in spleens of mice after different treatments. 6: aPDL1+DOX+CpG; 7: aPDL1/DOX@DNA Gel.

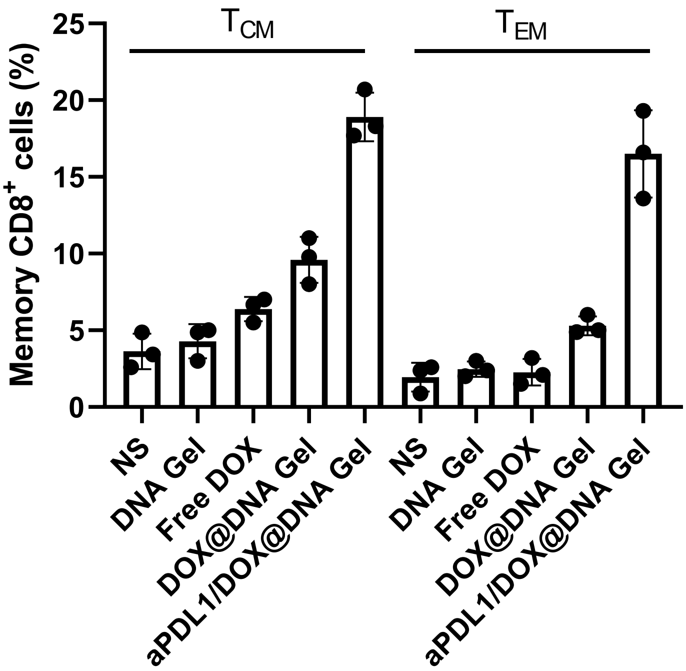

Figure S34. Statistical analysis of T_{CM} and T_{EM} cells in spleens of mice after treatments with NS, DNA Gel, free DOX, DOX@DNA Gel, and aPDL1/DOX@DNA Gel, respectively, as shown in Figures S32 and S33. Of note, the capability for activating memory T cells follows the order of aPDL1/DOX@DNA Gel > DOX@DNA Gel > free DOX > DNA Gel > NS. Evidently, DOX synergizing with CpG in DNA Gel plays a pivotal role in the immune memory effect, while the immune memory effect can be further enhanced by the inclusion of aPDL1.

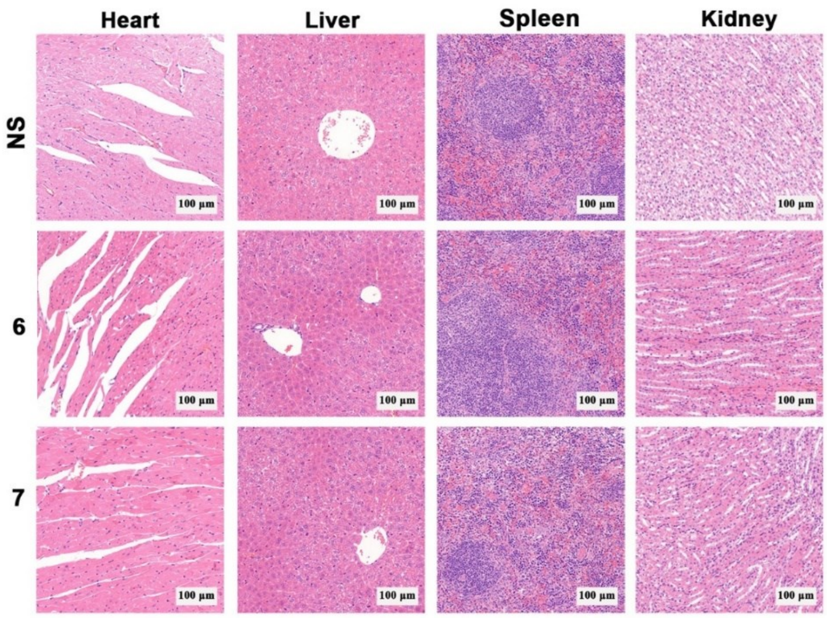

Figure S35. H&E staining of major organs including heart, liver, spleen, and kidney after different treatments. 6: aPDL1+DOX+CpG; 7: aPDL1/DOX@DNA Gel.

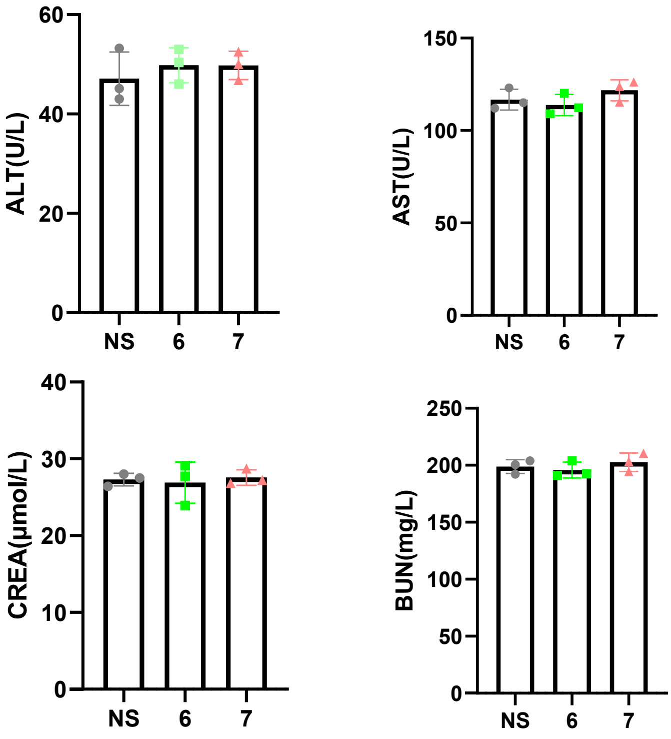

Figure S36. Blood biochemistry analysis of B16-tumor-bearing mice at the end of different treatments. The data are shown as mean ± SD (n = 3). 6: aPDL1+DOX+CpG; 7: aPDL1/DOX@DNA Gel.
